# Finding Multiple Optimal Solutions to an Integer Linear Program by Random Perturbations of Its Objective Function

**DOI:** 10.3390/a18030140

**Published:** 2025-03-04

**Authors:** Noah Schulhof, Pattara Sukprasert, Eytan Ruppin, Samir Khuller, Alejandro A. Schäffer

**Affiliations:** 1 Cancer Data Science Laboratory, National Cancer Institute, National Institutes of Health, Bethesda, MD 20892, USA; 2 Department of Computer Science, Northwestern University, Evanston, IL 60201, USA; 3 Databricks Inc., San Francisco, CA 94105, USA

**Keywords:** integer linear program, selection problems, multiple optima, randomized algorithms, parallel algorithms

## Abstract

Integer linear programs (ILPs) and mixed integer programs (MIPs) often have multiple distinct optimal solutions, yet the widely used Gurobi optimization solver returns certain solutions at disproportionately high frequencies. This behavior is disadvantageous, as, in fields such as biomedicine, the identification and analysis of distinct optima yields valuable domain-specific insights that inform future research directions. In the present work, we introduce MORSE (Multiple Optima via Random Sampling and careful choice of the parameter Epsilon), a randomized, parallelizable algorithm to efficiently generate multiple optima for ILPs. MORSE maps multiplicative perturbations to the coefficients in an instance’s objective function, generating a modified instance that retains an optimum of the original problem. We formalize and prove the above claim in some practical conditions. Furthermore, we prove that for 0/1 selection problems, MORSE finds each distinct optimum with equal probability. We evaluate MORSE using two measures; the number of distinct optima found in r independent runs, and the diversity of the list (with repetitions) of solutions by average pairwise Hamming distance and Shannon entropy. Using these metrics, we provide empirical results demonstrating that MORSE outperforms the Gurobi method and unweighted variations of the MORSE method on a set of 20 Mixed Integer Programming Library (MIPLIB) instances and on a combinatorial optimization problem in cancer genomics.

## 1. Introduction

An integer linear program (ILP) or a mixed integer program (MIP) may have multiple distinct optimal solutions. Typically, ILP solvers, such as Gurobi, SCIP, and CPLEX, find the optimal value of the objective function and one optimal solution. In the present work, we implement and test MORSE (Multiple Optima via Random Sampling and careful choice of the parameter Epsilon), an efficient and parallelizable algorithm to generate distinct optima for ILPs and MIPs with multiple optimal solutions that differ in at least one of the variables constrained to be integral. An example of a necessarily linear objective function for an ILP is 3.2x+1.5y. The variables x and y must take on integer values, but the coefficients 3.2 and 1.5 and the value of the objective function need not be integers. The MORSE algorithm maps multiplicative perturbations to the coefficients on binary and integer variables in the objective function, while keeping all model constraints unchanged. The intuition is that the random perturbation breaks ties among the original optimal solutions, determining which solution(s) are favored in the perturbed problem.

### 1.1. Problem Motivation

This project was primarily motivated by a problem in cancer genomics for which we implemented the software MadHitter, version 1 [1], which uses Gurobi or SCIP to solve variations of the classical combinatorial optimization problem *minimum hitting set* [2]. Taking as input single-cell mRNA data from patient tumors, MadHitter finds an optimal combination of target genes to treat either an individual patient or a cohort of multiple patients, and suggests different genes and their encoded proteins as future targets for cancer drug development. For the modeling in MadHitter, we treat each targetable gene equivalently, such that any solution targeting g genes is advantageous to any solution targeting g+1 genes, regardless of what may be known about the genes. MadHitter does not choose between two optimal gene sets of the same minimum size. Clinically, most genes cannot be targeted by existing drugs, suggesting that solutions with more “druggable” genes are preferable. Moreover, biochemists and drug developers have expert knowledge as to which genes will be easier to target chemically with future drugs. Therefore, they are interested in identifying multiple optimal solutions to choose a solution containing genes that may be targeted for the treatment of future patients.

Gurobi offers a solution pool method that finds multiple optimal solutions of a linear program. Technically, if model stores the Gurobi problem instance, then one specifies the intent to collect multiple solutions with a command such as:
model.setParam(GRB.param.PoolSolutions, num_solutions)
and then one retrieves the $i^{\text{th}}$ solution with a command such as:
select_solution(model, i).

We tested the solution pool method within MadHitter, but regrettably found that many solutions were frequently missed when sampling 50–100 optimal solutions. Many of the optima returned were ‘copies’ of the same optimal hitting sets, differing only in the values of variables that appear exclusively in the model constraints. However, only solutions that differ in the objective function variable values are of interest for MadHitter. Indeed, in most other MIP applications, only the variables in the objective function have practical importance for decision making. Variables that appear only in the constraints are typically needed to formulate the problem rigorously and precisely as an MIP. If one is interested in distinguishing solutions that differ in constraint-only variables, one can add the constraint-only variables to the objective function multiplied by tiny coefficients, so that they are effectively lower-order terms. The difficulties in using solution pool motivated us to design and implement a novel method, MORSE, to generate distinct optimal solutions.

There are two available methods that aim to find all optimal solutions of integer programs. Both methods modify the use of the branch-and-bound tree in finding a single optimal solution. The unrestricted subtree method is implemented via the functions SCIPcount and SCIPgetNCountedSols in SCIP [3]; the first function finds the distinct solutions, and the second function returns the number of distinct solutions found. The SCIP documentation provides more information on these functions. The one-tree method is implemented via the function populate in CPLEX [4]. Associated with the function is a parameter denoted by either CPX_PARAM_MIP_POOL_CAPACITY, SolnPoolCapacity, or PopulateLim (depending on the programming language interface) that sets an upper bound on the number of solutions stored. The function populate allows the user to specify that suboptimal solutions within some percentage of the optimal objective function value should also be returned. For more information, see the CPLEX documentation. Both methods are inherently sequential, and the solutions are unavailable until the respective functions have fully completed their execution. Both methods are yet to be implemented in Gurobi, as indicated by the current Gurobi documentation (accessed on 31 December 2024).

Closely related works seek to identify a set of k diverse solutions that are optimal (or near-optimal) and maximize some measure of diversity. The most popular measure of diversity is the sum of the Hamming distances between the values of the integer variables [5]. We explain the Hamming distance measure and an alternative in Section 2. Danna and Woodruff [5] formulated the problem of finding k diverse near-optimal solutions, executing the CPLEX one-tree method for a fixed duration to generate a large set S of near-optima. From the set S, the [5] exact method selects the most diverse set of k solutions. Danna and Woodruff also proposed a local search heuristic method for instances for which the aforementioned method is excessively slow [5]. Trapp and Konrad [6] proposed a different method to find k optima for 0–1 ILPs. This method produces solutions one at a time, such that solution s+1 is maximally diverse with respect to the previously produced s solutions for s≥1. An advantage of this sequential method is that one can examine solutions one at a time and decide whether to continue. A disadvantage is that the method does not handle general integer variables. In sum, two gaps in the previous work are that Gurobi lacks a method to find all optimal solutions, and the previous methods in SCIP and CPLEX are inherently sequential. In the present work, we tested primarily with Gurobi and secondarily with SCIP and CPLEX.

### 1.2. Additional Literature Review

In biomedicine, including cancer genomics, researchers using ILPs have repeatedly been interested in finding multiple optimal solutions [7–9]. In biology, understanding the diversity of optimal solutions can yield valuable insights [7]. For some biological problems, there is specialized knowledge that renders certain solutions preferable to others for reasons that are not represented in the objective function [10,11]. Biomedical papers (some describing named scientific software packages) that describe methods to find multiple optima for ILPs or MIPs include MedØlDatschgerl by [8,12,13], MTM by [9,14] CellNetAnalyzer by [15], Rosetta by [16], OptCouple by [17,18], Corex by [10], DEXOM by [19–21], OCSANA by [22], and MCSEnumerator by [11]. Several studies also exist on non-biomedical topics including energy management [23], materials science [24], computer chip layout [25], user interface design [26], urban planning [27], and clustering [28], which attempted to enumerate all optima for similar reasons.

Because of the problem of finding the cost of any optimal solution to ILPs is NP-complete ([29] Chapter 2), it follows from computational complexity theory that the problem of counting the number of solutions to an ILP is #P-complete ([30] Section 7.3). Nevertheless, as shown below, there are practical ILPs and MIPs for which the number of optimal solutions is >1 but is not exceedingly large. We tested MORSE on such instances.

The problem of finding all optimal solutions to an ILP has been studied since the seminal paper of Balas and Jeroslow [31]. They restricted attention to 0–1 ILPs, also known as *selection problems*, in which all variables are constrained to the values 0 and 1, representing decisions to select or exclude each variable. Examples include facility location and minimum hitting set. Their key insight was that each time a new optimum is found, one can add a constraint, known as a *cut*, to the ILP, so that the newly found optimal solution is no longer feasible in the adjusted problem, but all other optimal solutions remain both feasible and optimal. This yielded an effective, sequential method to enumerate optimal solutions, well-suited to the computers available in 1972. This method was later generalized to ILPs with integer-valued variables by [32]. Our approach perturbs the objective function, rather than modifying the constraints, and is better suited to parallel computers that are now available.

To our knowledge, the first paper to propose randomization for the problem of finding multiple optima was [33]. Few subsequent papers have investigated randomization in this context; one such study is that of [6], in which a small experiment found a randomized method inferior to deterministic methods. The infrequent use of randomization for ILPs contrasts with linear programming, for which randomization has been essential to sensitivity analysis [34].

Sensitivity analysis, also called post-optimality analysis, of mathematical programs concerns the extent to which the coefficients or right-hand sides of constraints can be varied while retaining optimality. Here, we are instead concerned with finding tiny perturbations that break ties between optimal solutions without introducing new optima, though some informal connections between our work and past work on sensitivity analysis are worth noting.

The topic of sensitivity analysis for MIPs was reviewed by [35] and more recently by [36]. One general style of sensitivity analysis for MIPs that evaluates small perturbations is the *tolerance approach*, pioneered by Wendell [37] and in many related papers by him and his colleagues. In the tolerance approach, one question that has been studied by [36,38–40] is the extent to which one can vary one or more coefficients in an MIP or ILP with a unique optimal solution while preserving the optimality of an optimal solution to the unperturbed problem. The resulting interval (for one coefficient) or region (for more than one coefficient) is called the *tolerance region*. One general negative result is that for 0/1 MIPs it is NP-hard to determine the tolerance region of a single coefficient in the objective function [38].

There also exist some positive results on varying one or two coefficients. For example, Ref. [36] formulated the problem of finding the tolerance interval/region for one or two coefficients as an MIP itself. In the special case where the instance is a knapsack ILP, Ref. [40] provided a polynomial-time algorithm to find the tolerance interval for each individual coefficient.

### 1.3. Use of MIPLIB for Testing

To help gauge and stimulate progress in the ILP field, experts, beginning in 1992, have curated MIPLIB, a library of publicly available MIP problems. There efforts were inspired by NETLIB, a similar library for linear programs. The technical report on MIPLIB version 3.0 [41] explains the motivation to develop MIPLIB. Most recently updated in 2017, the current MIPLIB 6.0 set contains 1065 problems [42]. All MIPLIB instances are assigned alphanumeric names, which may be descriptive (such as protfold, a protein folding problem from the MIPLIB 2010 set [43]) or not descriptive (such as 30_70_45_05_100, described as “Geographic radar station allocation” in the MIPLIB 2017 set [42]). The aforementioned studies of [3–6] used MIPLIB to test their respective methods.

### 1.4. Our Contributions

In Section 2, we present the theory of our perturbation method and the evaluation metrics we use to test it. We implemented our method on top of the Gurobi MIP solver, and compared it to possible usages of the Gurobi solution pool method, the SCIP count method, and the CPLEX populate method. We tested MORSE largely on 20 published MIP instances. A sample experiment compares how many distinct optimal solutions are observed after r independent Gurobi runs, both with existing methods and with our method.

We evaluate the diversity of the solutions produced by MORSE, though the algorithm does not explicitly maximize the diversity, as was achieved by [5,6]. In Section 3, we show that our perturbation method outperforms Gurobi’s solution pool on MIPLIB and MadHitter instances, as quantified by the diversity metrics defined in Section 2 and by the number of distinct optima found. We conclude in Section 4 with a summary of our empirical results, some limitations, and some possible future directions.

## 2. Materials and Methods

### 2.1. Perturbing the Coefficients in the Objective Function

We present an algorithm, MORSE, to map random perturbations to the coefficients on the binary and integer objective function variables to efficiently find distinct optima for ILP instances with multiple optima. One theory, described below, assumes that variables are restricted to be integers, though our implementation allows continuous variables without perturbing the coefficients of continuous variables. In either case, MORSE always returns an optimal solution. When there are continuous variables, the MORSE software, version 1 checks that optimality is preserved. We refer to the Gurobi single-solution or solution pool method as the conventional *homogeneous/uniform weights method*, in which we solve the problem in its original, unmodified form. In particular, Gurobi does not alter the original coefficients of binary or integer variables in the objective function. In practice, one can obtain multiple solutions via the homogeneous weights method by varying the Gurobi random seed and/or by using the solution pool. In our experiments, we varied the random seed and obtained one solution per Gurobi run to facilitate a head-to-head comparison with MORSE. We use the term *homogeneous/uniform weights* because leaving the original coefficients unchanged is equivalent to multiplying all the coefficients by 1 homogeneously or uniformly.

Central to MORSE is the selection of a small value ε>0 that determines the maximum extent to which the coefficients may be varied, as explained first with an example and subsequently with formulae.

Consider the following instance:

$$\begin{array}{cc} \text{maximize} & x_{1}+x_{2}\\ \text{subject}\;\text{to}\;\text{the}\;\text{constraints} & x_{1},x_{2}\leq 2,\\ & x_{1}+x_{2}\leq 3.5,\\ & x_{1},x_{2}\in Z. \end{array}$$

The objective function can be expressed as $c^{T}x$, where c=11 and x=x1x2. Suppose we choose ε=0.01. Next, we generate a random *perturbation vector* $v\overset{iid}{\sim }U(1-\varepsilon ,1+\varepsilon )=U(0.99,1.01)$. If all variables are integral, then dim(v)=dim(x), which is the number of variables in the objective function. If there are continuous variables, dim(v) is the number of binary or integer variables (defined formally below).

An example of such a perturbation vector is v1=0.998641.00142.

We subsequently define the perturbed coefficient vector c1′=v1Tc=0.998641.00142.

This yields the perturbed objective function ${c^{\prime }}_{1}^{T}x=0.99864x_{1}+1.00142x_{2}$. Without modifying the constraints, we solve the resulting instance and obtain the optimum $\left\{x_{1}=\right.\left.1,x_{2}=2\right\}$.

On another execution of MORSE, with the same value for ε, imagine that we generate the perturbation vector v2=1.000450.99312. Following the same steps as above, we obtain the optimum $\left\{x_{1}=2,x_{2}=1\right\}$.

By executing two independent runs of MORSE, we can find the two distinct optima for the provided instance. To generalize this notion, for any instance with s>1 distinct optima, we can try to find all distinct optima in r≥s independent MORSE runs.

### 2.2. Choosing ε

Let P be a MIP with objective function $\sum_{i=1}^{n}\;c_{i}x_{i}$ such that $c_{i}\neq 0$ and $x_{i}\in Z$ for at least one index i. We assume that each variable has type vtype(x), which is one of three possible types: *Binary, Integer*, or *Continuous*. In MIPLIB instances, each variable x has specified lower and upper bounds that we denote by lb(x), ub(x) respectively. The MadHitter instances we used for testing are ILP selection problems in which every variable in the objective function is of type *Binary* with lower bound 0 and upper bound 1.

Define $S=\sum_{i=1}^{n}\;f\left(c_{i},x_{i}\right)$ where

$$f(c,x)=\left\{\begin{array}{ll} |c|\cdot \text{max}(|\text{lb}(x)|,|\text{ub}(x)|), & \text{vtype}(x)\neq \text{Continuous},\\ 0, & \text{vtype}(x)=\text{Continuous}, \end{array}\right.$$

Then $\varepsilon =\frac{1}{2S}$.

For example, suppose the objective function is 2x+3y+z, all variables are of type *Integer*, and the variables are constrained to be in the following intervals x∈[-2,1], y∈[1,4], z∈[-4,2]. Then, S=(2×2)+(3×4)+(1×4)=20, and ε=1/40=0.025. If we scale the coefficients up by a factor of 5 to make the objective function 10x+15y+5z, then S=100 and ε=1/200=0.005, decreasing by a factor of 5. If we scale the coefficients down by a factor of 5 to make the objective function 0.4x+0.6y+0.2z, then S=4 and ε=1/8=0.125, increasing by a factor of 5. In general, scaling the objective function coefficients by a factor of k>0 scales ε by a factor of 1/k.

An advantage of this method is that it can be executed by simply examining the structure of the instance. If variable bounds are not available, then one can solve the instance to obtain a single optimum and use the absolute values of the variables in that solution to define ε, which can then be used to search for other optima.

### 2.3. Applying the Perturbation, Pseudocode for MORSE

Define perturbation vector $v_{1},\ldots ,v_{n}\overset{iid}{\sim }U(1-\varepsilon ,1+\varepsilon )$. Define $P^{\prime }$, the perturbed instance of P, as an MIP with identical constraints as P (as defined in Section 2.2) and objective function

$$\sum_{i=1}^{n}{c^{\prime }}_{i}x_{i}=\sum_{i=1}^{n}g\left(c_{i},v_{i},x_{i}\right)$$

where

$$g(c,v,x)=\left\{\begin{array}{ll} cvx, & \text{vtype}(x)\neq \text{Continuous},\\ cx, & \text{vtype}(x)=\text{Continuous}. \end{array}\right.$$

The full pseudocode for MORSE is shown below with Algorithms 1 and 2 as helper functions used in Algorithm 3.


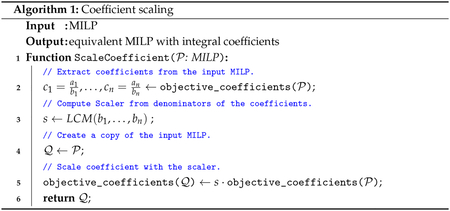



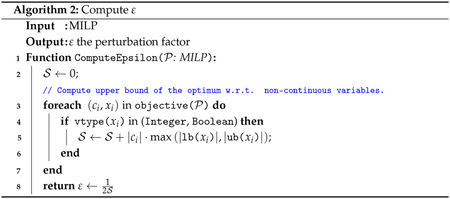



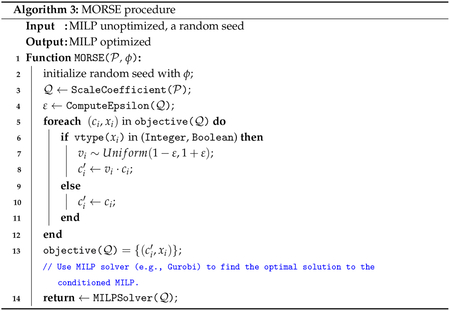


The Python implementation (hosted at https://github.com/ruppinlab/MORSE, accessed from mid-2023 onward and most recently on 2 March 2025) consists of three top-level scripts (solve.py, parallel.py, agg.py) designed for practical usage and an additional script (morse.py) of helper functions. The primary script, solve.py, solves a single instance one time. To execute several runs in parallel, parallel.py generates parallelizable bash scripts, which can subsequently be executed to produce multiple optimal solutions. agg.py aggregates these solutions into a single file, and the helper script morse.py implements the MORSE procedure as specified in Algorithm 3. Since MORSE is built on the Gurobi Optimizer, a valid Gurobi license is required.

### 2.4. Some Theory to Justify the Choice of ε

Our intuition is that by making ε sufficiently small, any optimum with the perturbed objective function will also be an optimum for the original objective function. We prove that optima are preserved for ILPs with integer coefficients in Theorem 1. However, the converse is untrue; for any specific perturbation of the objective function, only some optima for the original problem may remain optima for the perturbed problem, as shown in the example above, in which a perturbation preserves one possible optimum, but not the other. Our intuition is that some partial converse holds such that, for every optimum of the unperturbed problem, there exist perturbations that retain that optimum; this intuition is partly formalized in Theorems 2 and 3. When there are continuous variables, however, our implementation checks that optima are conserved, because this cannot be guaranteed.

**Theorem 1.** *Suppose all the coefficients in the original problem are integers, all the variables are binary or integer, and the problem* P *is a maximization problem. There exists a sufficiently small* ε>0 *such that for any perturbation* $c_{1},\ldots ,c_{n}\to {c^{\prime }}_{1},\ldots ,c_{n}^{\prime }$ *and any resulting optimum* ${x^{\prime }}_{1},\ldots ,{x^{\prime }}_{n}$ *of the perturbed instance* $P^{\prime },{x^{\prime }}_{1},\ldots ,{x^{\prime }}_{n}$ *is also an optimum of the original instance* P.

**Proof.** Let $x_{1},\ldots ,x_{n}$ be an optimum of P. Define $\varepsilon <{\left(2\sum_{i=1}^{n}\left|c_{i}x_{i}\right|\right)}^{-1}$ and let $P^{\prime }$ be the perturbed instance of P with respect to ε. Consider ${x^{\prime }}_{1},\ldots ,{x^{\prime }}_{n}$ such that ${x^{\prime }}_{1},\ldots ,{x^{\prime }}_{n}$ is optimal for $P^{\prime }$ but is not optimal for the original instance P. Due to the integrality constraints, the assumption that the coefficients are integers, the assumption that the variables are not continuous, and the assumption that the problem is a maximization problem,

(1) $$P\left(x_{1},\ldots ,x_{n}\right)-P\left(x_{1}^{\prime },\ldots ,x_{n}^{\prime }\right)\geq 1.$$

Consider what happens to these values in the perturbed problem $P^{\prime }$. Seeking a contradiction, suppose that $x^{\prime }$ has a better objective function value than x for $P^{\prime }$, then

(2) $$P^{\prime }\left(x_{1}^{\prime },\ldots ,x_{n}^{\prime }\right)-P^{\prime }\left(x_{1},\ldots ,x_{n}\right)\leq \left[P\left({x^{\prime }}_{1},\ldots ,x_{n}^{\prime }\right)-P\left(x_{1},\ldots ,x_{n}\right)\right]+\left[\left|P^{\prime }\left(x_{1},\ldots ,x_{n}\right)-P\left(x_{1},\ldots ,x_{n}\right)\right|\right]+\left[\left|P^{\prime }\left({x^{\prime }}_{1},\ldots ,x_{n}^{\prime }\right)-P\left({x^{\prime }}_{1},\ldots ,{x^{\prime }}_{n}\right)\right|\right]\;<-1+\frac{1}{2}+\frac{1}{2}\;<0$$

Because we assumed the original problem P as a maximization problem, this is a contradiction. The first term in square brackets is at most −1 based on Equation (1). The second and third terms in square brackets are <1/2 due to the definition of ε. □

In our experiments, most instances are minimization problems, due to the structure of MIPLIB, so we must also consider the complementary case in which P is a maximalization problem. For the latter case, the first numbered inequality (Equation (1)) becomes, instead

$$P\left(x_{1}^{\prime },\ldots ,x_{n}^{\prime }\right)-P\left(x_{1},\ldots ,x_{n}\right)\geq 1$$

and the subsequent contradiction (Equation (2)) is that

$$P^{\prime }\left(x_{1},\ldots ,x_{n}\right)-P^{\prime }\left(x_{1}^{\prime },\ldots ,x_{n}^{\prime }\right)<0$$

meaning that the original solution has a lower (better for minimization) objective function value than the alternative solution.

The assumption that all the coefficients for the integer-constrained variables are themselves integral does not hold for some MIPLIB instances. The idea behind the proof of Theorem 1 can be generalized to fractional coefficients by scaling up the problem by multiplying all the coefficients by the least common multiple of the denominators. For example, if the objective function is 1.3x+3.2y+2.1z, which is equivalent to $\frac{13}{10}x+\frac{16}{5}y+\frac{21}{10}z$, and the least common multiple of the denominators is 10. We can scale up the objective function to 13x+32y+21z to make the adjusted coefficients integral and then, Theorem 1 applies to the adjusted problem. As shown in the examples above in Section 2.2, the consequence of multiplying the coefficients by 10 is to divide ε by 10. The scaling procedure is specified by Algorithms 1. Preservation of optima after perturbing coefficients for continuous variables cannot be guaranteed because the difference in objective function values for two solutions could be infinitesimally small. When there are continuous variables, MORSE checks that potential solutions are optimal (to within machine tolerance) for the original problem.

For the proof of Theorem 1, we assumed that one optimal solution is available and we used the bound $\varepsilon <{\left(2\sum_{i=1}^{n}\left|c_{i}x_{i}\right|\right)}^{-1}$ without assuming any bounds on the variables $x_{i}$. If upper and lower bounds on variables are available, as is the case for MIPLIB, then we can instead use the formulation in Section 2.2 and the same reasoning applies. Using the available bounds on the variables simplifies MORSE because one does not need a sequential phase of finding one optimal solution before the subsequent phase of running multiple parallelized runs to obtain multiple optimal solutions.

Next, we prove, in theory, that MORSE can find any optimal solution.

**Theorem 2.** *For any optimum* $x_{1},\ldots ,x_{n}$ *of* P, *there exists a perturbation* ${c^{\prime }}_{1},\ldots ,{c^{\prime }}_{n}$ *such that $x_{1},\ldots ,x_{n}$ is also an optimum of $P^{\prime }$*

**Proof.** Choose the identity perturbation such that ${c^{\prime }}_{1}=c_{1},\ldots ,{c^{\prime }}_{n}=c_{n}$. Then $P=P^{\prime }$ and optima are conserved. □

Next, we consider the effects of non-identity perturbations in breaking ties.

**Theorem 3.** *Suppose the instance* P *is defined as in Theorem 1. For any optimum $x_{1},\ldots ,x_{n}$ of* P, *there exists a perturbation ${c^{\prime }}_{1},\ldots ,{c^{\prime }}_{n}$ such that* ${c^{\prime }}_{1}\neq c_{1},\ldots ,{c^{\prime }}_{n}\neq c_{n}$ *and* $x_{1},\ldots ,x_{n}$ *is also an optimum of $P^{\prime }$*.

**Proof.** Let ε be defined as in the proof of Theorem 1. Define ${c^{\prime }}_{i}=\left\{\begin{array}{ll} (1+\varepsilon )\cdot c_{i}, & c_{i}x_{i}>0,\\ (1-\varepsilon )\cdot c_{i}, & c_{i}x_{i}<0. \end{array}\right.$

If the values of $x_{i}$ remain unchanged, then the constraints of $P^{\prime }$ remain satisfied. Moreover, any solution that satisfies the constraints of P also satisfies the constraints of $P^{\prime }$. By this perturbation, ${c^{\prime }}_{1}x_{1}+\ldots +{c^{\prime }}_{n}x_{n}>c_{1}x_{1}+\ldots +c_{n}x_{n}$. Hence, because of the choice of ε, the absolute net change in the objective function value, which is given by $\varepsilon \sum_{i=1}^{n}\left|c_{i}x_{i}\right|$, is less than 1.

Aiming to find a contradiction, suppose that some other solution ${\overset{ˆ}{x}}_{1},\ldots ,{\overset{ˆ}{x}}_{n}$ yields a higher objective function than $x_{1},\ldots ,x_{n}$. Consider the difference in objective function value of this solution for the original instance P and the perturbed instance $P^{\prime }$. For each of the k terms involving an integer-constrained variable, if $c_{i}{\overset{ˆ}{x}}_{i}>0$, then $\left|c_{i}{\overset{ˆ}{x}}_{i}-{c^{\prime }}_{i}{\overset{ˆ}{x}}_{i}\right|\leq \varepsilon c_{i}{\overset{ˆ}{x}}_{i}$. Therefore, the net increase in objective function value for ${\overset{ˆ}{x}}_{1},\ldots ,{\overset{ˆ}{x}}_{n}\leq \varepsilon \sum_{i=1}^{n}\left|c_{i}x_{i}\right|$. Thus, the solution ${\overset{ˆ}{x}}_{1},\ldots ,{\overset{ˆ}{x}}_{n}$ cannot have a strictly better objective function value in the permuted problem $P^{\prime }$ than does the original solution $x_{1},\ldots ,x_{n}$. □

From Theorem 3, it follows that the probability of finding any particular optimal solution is >0. Nevertheless, the result is weak because the volume around the optimal solution in the proof depends on ϵ, and hence, inversely on the coefficients. Next, we prove in two steps a stronger result about MORSE’s capability to find different optima for the special case of 0–1 selection problems.

**Lemma 1.** *Let* P be a *0/1 ILP that is a selection and minimization problem with objective function* $\sum_{i=1}^{n}\;c_{i}x_{i}$, *and every coefficient $c_{i}=1$*. *Suppose one perturbs the objective function by multiplying each coefficient by some $p_{i}\sim U(1-\varepsilon ,1+\varepsilon )$ as the* MORSE *method does to arrive at the new problem $\sum_{i=1}^{n}\;p_{i}x_{i}$*. *Then, any optimal solutions $\left\{x_{j_{1}},x_{j_{2}},\ldots ,x_{j_{d}}\right\}$ to the perturbed problem are optimal solutions to the original problem. The indices of the selected variables have the additional property that the sum of perturbed coefficients $\sum_{k=1,d}\;p_{j_{k}}$ corresponding to the variables that are selected is minimized among all possible sums of d perturbed coefficients*.

**Proof.** Since P is a selection problem, there must be some positive integer value d, such that all optimal solutions are of size d. By Theorem 1, any optimal solution to the perturbed problem is a solution to the original selection problem. Suppose the variables that are set to 1, and hence, selected in an optimal solution to the original problem are $\left\{x_{j_{1}},x_{j_{2}},\ldots ,x_{j_{d}}\right\}$. Then, the objective function value of that solution in the original problem is d and the objective function value of that solution in the perturbed problem is $\sum_{k=1}^{d}p_{j_{d}}$. Any solution that assigns 1 to exactly d variables in the perturbed problem will have an objective function value that is the sum of the perturbed coefficients for the d selected variables. Therefore, the optimal solutions of the perturbed problem are precisely those that minimize $\sum_{k=1}^{d}p_{j_{d}}$. It is possible that multiple solutions to the perturbed problem are tied for the minimum sum, although this is unlikely in practice. □

**Theorem 4.** *Let* P *be a 0/1 ILP that is a selection, minimization problem with objective function* $\sum_{i=1}^{n}\;c_{i}x_{i}$, *and every coefficient $c_{i}=1$*. *Suppose one perturbs the objective function by multiplying each coefficient by some* $p_{i}\sim U(1-\varepsilon ,1+\varepsilon )$ *as the MORSE method does. Suppose (as explained in the Proof of Lemma 1) that all optimal solutions have objective function value* d *meaning that exactly* d *variables have value 1. Suppose $\left\{x_{j_{1}},x_{j_{2}},\ldots ,x_{j_{d}}\right\}$ and* $\left\{x_{k_{1}},x_{k_{2}},\ldots ,x_{k_{d}}\right\}$ *are two distinct optimal solutions of the original problem. Then, the probabilities that* $\left\{x_{j_{1}},x_{j_{2}},\ldots ,x_{j_{d}}\right\}$ *and* $\left\{x_{k_{1}},x_{k_{2}},\ldots ,x_{k_{d}}\right\}$ *are optimal solutions of one problem perturbation from MORSE are equal*.

**Proof.** Let $M_{j}$ be the set of perturbations that make $V_{j}=\left\{x_{j_{1}},x_{j_{2}},\ldots ,x_{j_{d}}\right\}$ optimal for the perturbed problem. Let $M_{k}$ be the set of perturbations that make $V_{k}=\left\{x_{k_{1}},x_{k_{2}},\ldots ,x_{k_{d}}\right\}$ optimal for the perturbed problem. We show constructively that there is a one-to-one correspondence $C:M_{j}\to M_{k}$. It follows that the probability that a uniformly randomly selected perturbation is in $M_{j}$ equals the probability that a uniformly randomly selected perturbation is in $M_{k}$. Let $p_{j}$ be a perturbation of coefficients in $M_{j}$. We construct a corresponding perturbation $p_{k}$ in $M_{k}$. Specifically, the function C maps $p_{j}$ to $p_{k}$ by permuting indices without changing the set of uniformly selected multipliers. For example, suppose there are three variables and $p_{j}$ perturbs the coefficients by multiplying $c_{1}$ by 0.9998, $c_{2}$ by 1.0007, and $c_{3}$ by 0.9993. Then, an example of a permuted perturbation would be instead to multiply $c_{1}$ by 1.0007, to multiply $c_{2}$ by 0.9993 and $c_{3}$ by 0.9998. For i=1,…n, we have two cases:

1. If $x_{i}$ is the rth variable $x_{jr}$ in $V_{j}$, then C maps the multiplier of $c_{i}=c_{jr}$ to be instead the multiplier of $c_{kr}$.
2. If $x_{i}$ is the sth variable absent from $V_{j}$, then C maps the multiplier of $c_{i}$ to be instead the multiplier for the coefficient of the sth variable absent from $V_{k}$, preserving the value of the perturbed coefficient.

Since the mapping C preserves the values of the coefficients and of the objective function in the perturbed problem, it follows from Lemma 1 that C preserves optima of the perturbed problem. Since C preserves the values of the variables and any permutation of the dimensions is a one-to-one correspondence on the set of dimensions, it follows that C is a one-to-one correspondence between $M_{j}$ and $M_{k}$. □

We are also interested in the probability that each possible optimum of the original problem appears as an optimum of a perturbed problem. Suppose there are v variables that appear in the objective function, each corresponding to a distinct term. Thus, it follows that the space of perturbations is isomorphic to the hypercube $𝒬:=[1-\varepsilon ,1+\varepsilon {]}^{v}$. The hypercube representation is reminiscent of the work of [31] on multiple optima for binary programs, though there exist a few notable differences. Their work focused exclusively on the vertices of the hypercube, each of which represented a different solution; 0 for the non-selected variables and 1 for the selected variables. By contrast, our interest extends to both the boundary and interior of 𝒬, where 𝒬 instead represents perturbations to the original objective function, rather than solutions. For 𝒬, “boundary” refers to any point at which any one of the dimensions takes on either of the values 1-ε or 1+ε, and “interior” refers to all other points. Whether a perturbation is on the boundary or in the interior does not affect the operation of MORSE. In our development and testing, we found it useful to test perturbations on the boundary to gain confidence in the correctness and robustness of the MORSE code.

Employing the hypercube formalism, one may ask:

**Question 1.** *For any perturbation, which solutions remain optimal?*

**Question 2.** *Under what conditions on the perturbation* p∈𝒬 *is the solution to the perturbed problem for* p *unique?*

**Question 3.** *For any optimum* x *to the original, unperturbed problem, does there exist a perturbation* p, such that if one applies p, *then* x *remains an optimum to the perturbed problem? (We gave an affirmative answer under some assumptions in Theorem 3; it would be desirable to determine if these assumptions can be weakened.)*

*(a) If so, we can define* V(x) *as the volume within the hypercube of perturbations that preserve the optimality of* x. Can one determine the upper and lower bounds on V(x)?

Our intuition, which we tested empirically via the solution pool method, suggests that for the majority of perturbations, the solution to the perturbed problem is unique for the variables constrained to be integers. However, there must be boundary regions of zero volume at which two or more solutions are tied. In Theorem 4, we answered Question 3(a) affirmatively for selection problems. We hypothesize that for some subset of the problems we consider, the answer to Question 3 is positive, and this is verifiable for MIPLIB instances for which the number of distinct optima is known. If all optima of the unperturbed problem are identified, then one can use repeated sampling of perturbations to empirically estimate the probability that each optimal solution is found.

### 2.5. Conceptual Comparison Between MORSE and a Previously Tested Perturbation Method

Our perturbation method differs from a similar method originally proposed by [34] in the context of linear programming and later implemented for binary/integer problems by [6]. In the latter work, each binary and integer coefficient in the objective function is multiplied by the quantity $\left(1+\varepsilon \xi_{ij}\right)$, where ε is a positive constant and $\xi_{ij}\overset{iid}{\sim }U(-1,1)$. In both cited studies, the choice of ε did not depend on the input instance. As a consequence, optima may not be preserved; indeed, the fact that small perturbations can change optimal solutions was a key point on non-robustness that Ben-Tal and Nemirovski made in their perturbation analysis. Trapp and Konrad [6] tested their version of a perturbation method on just three instances, and only for *fixed* values of ε, and found that the results were not favorable for finding all optimal solutions. Trapp and Konrad did not prove that their perturbation preserved optimal solutions, although their intent was to preserve optima or near-optima. In contrast, our MORSE method multiplies the coefficient for each binary and integer variable by a random real number in the range [1-ε,1+ε], where ε is determined *adaptively* based on the size of the coefficients in the objective function and the bounds on the objective function variables (see Section 2.2). As shown above, the perturbed instances generated by MORSE preserve optimal solutions.

### 2.6. MIPLIB as a Source of Instances for Testing

We downloaded select instances from MIPLIB to test our weighted perturbation method on a diverse set of optimization problems. We chose instances from the following overlapping lists:

1. Instances mentioned by Danna and colleagues in their 2007 study [4]
2. Instances mentioned by Danna and Woodruff [5]
3. Instances mentioned by Trapp and Konrad [6]
4. Instances that include binary and/or integer decision variables and classified as “easy” on MIPLIB (solvable in one hour or less by a commercial solver on default settings). Instances formally retired prior to the MIPLIB 2017 set (for which the task of finding one optimum had become “too easy”) were still considered for our testing, as the task of solving for multiple/all optima remains interesting and sufficiently complex. Such instances were obtained from the MIPLIB 1996 [41], 2003 [44], or 2010 [43] sets.
  To be considered for our central testing stage, instances needed to meet the following additional criteria:
5. The objective function includes binary and/or integer variables.
6. The instance has multiple optima.
  To enforce Criterion 5, algorithmic testing was needed, and was performed as follows:
  - Iterate through the variable types for all variables in the objective function.
  - If at least one variable type is binary or integer, accept the instance. Otherwise, discard the instance.

The pseudocode is shown in Algorithm 4.


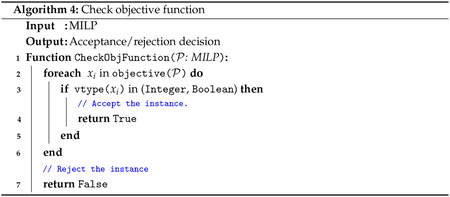


Criterion 6 could be partially evaluated by referencing the number of optima listed for each instance in [4]. Instances pulled from this paper were confirmed to have multiple optima as explained in Section 3. Additional testing was needed to confirm the existence of multiple optima for instances from [5] or [6] that did not intersect the list of instances in [4]. If our script detected that an instance did not satisfy any of the above criteria, the instance was dropped from further consideration.

### 2.7. Figures of Merit Used in Testing

As explained in Section 1, there are three freely available methods against which we can compare: the solution pool in Gurobi, the count function in SCIP, and the populate function in CPLEX. Since we implemented MORSE using Gurobi, most of our tests were executed in comparison to the solution pool method.

We compared the performance of our perturbation method against the standard, unperturbed method using the following test design. We executed r runs in parallel and obtained s optimal solutions, not necessarily distinct. We considered two figures of merit.

First, we examined the number of distinct optima found at least once among the s optimal solutions. Two solutions are considered distinct if and only if they differ in the value of at least one variable appearing in the objective function.

Second, we compared the solution sets on two previously used measures of diversity: *Hamming distance and Shannon entropy*. Solution set diversity has been previously quantified by average Hamming distance between solution vectors (see [5,6]).

**Definition 1** (Hamming distance). *For solution vectors* $x=\left[x_{1},\ldots ,x_{n}\right]$ *and* $y=\left[y_{1},\ldots ,y_{n}\right]$, *the Hamming distance* H(x,y) *is given by:*

$$H(x,y)=\sum_{i=1}^{n}f\left(x_{i},y_{i}\right)\;\text{where}\;f(x,y)=\left\{\begin{array}{ll} 1, & x\neq y\\ 0, & x=y \end{array}\right.$$

For example, consider solution vectors A=[1,2,3,4] and B=[1,0,3,5]; H(A,B)=2. The Hamming distance does not consider the magnitude of the difference between integer variables, and is increased by a uniform increment of 1 for each integer variable that differs between solutions.

**Definition 2** (Shannon entropy). *Let* $𝒳=\left\{x_{1},x_{2},\ldots ,x_{k\leq t}\right\}$ *be the set of solution vectors. The Shannon entropy is defined as follows*.


$$S(𝒳)=-\sum_{x\in 𝒳}p_{x}\;{\text{log}}_{2}\;\left(p_{x}\right)$$

In some other applications, the Shannon entropy is denoted by H, but we use the mnemonic S for the Shannon entropy and reserve the mnemonic H for the Hamming distance. For example, if there exist two solutions that are found with probabilities 1/3 and 2/3, then the Shannon entropy is as follows.


$$S(𝒳)=-\left(\frac{1}{3}{\text{log}}_{2}\;\frac{1}{3}+\frac{2}{3}{\text{log}}_{2}\;\frac{2}{3}\right)\approx 0.918$$

In our analysis, we are interested in the entire solutions as combinatorial objects. If instead there are three solutions and each solution occurs with probability 1/3, then the Shannon entropy of the solution set is as follows:

$$S(𝒳)=-3\left(\frac{1}{3}{\text{log}}_{2}\;\frac{1}{3}\right)\approx 1.585$$

Suppose, instead, we sample siux not necessarily distinct solutions, and we observe the distinct solutions A, B, C three times, two times, and one time, respectively. Then, the Shannon entropy of the solution multiset would be as follows:

$$-\left(\frac{1}{2}{\text{log}}_{2}\;\frac{1}{2}+\frac{1}{3}{\text{log}}_{2}\;\frac{1}{3}+\frac{1}{6}{\text{log}}_{2}\;\frac{1}{6}\right)\approx 1.459$$

We found one analysis of a problem-specific method to find multiple optima that used (higher) Shannon entropy on the values of individual variables among solutions as a figure of merit [10]. However, we did not find any previous study of multiple optima in MIPs that used Shannon entropy on the multiplicity of solutions, as in our study. In general, higher Hamming distance and higher Shannon entropy are preferable in that they indicate higher diversity. When comparing the Hamming distance or Shannon entropy for solution sets found by the two methods, we do not normalize by the number of distinct solutions found.

## 3. Results

We verified the correctness of the MORSE method and tested its performance empirically on the 20 MIPLIB instances listed in Table 1. For the first set of large-scale tests, in parallel, we executed 100 runs with MORSE and 100 Gurobi runs with homogeneous (unchanged) weights and collected one optimal solution for each run. For each problem instance, we assigned a distinct random seed to each run to maintain the reproducibility of the results, ensuring that the runs were independent and shared the same initial conditions. Runs were sorted using (as the sort key) either the random weights seed (for MORSE runs) or the Gurobi random generator seed (for homogeneous weights runs). From the two side-by-side orderings of homogeneous weights runs and MORSE runs, we aggregated lists of optima by taking one optimum from each run. Each list may contain duplicates because the runs were executed in parallel, in contrast to the inherently sequential approach of [31,32] described in Section 1.

We further ordered the paired lists of optima in three distinct ways:

1. *Seed-based*: Keeping the random number generator in seed-based ordering, as outlined above.
2. *Random shuffle*: Randomly shuffling the optima collected using the above seed-based order.
3. *Greedy for diversity*: Randomly choosing a solution from the seed-based list to be the first solution reported. Then, for each next solution chosen, choosing the solution that maximizes the average pairwise Hamming distance with all previously selected solutions.

**Figure 1.**
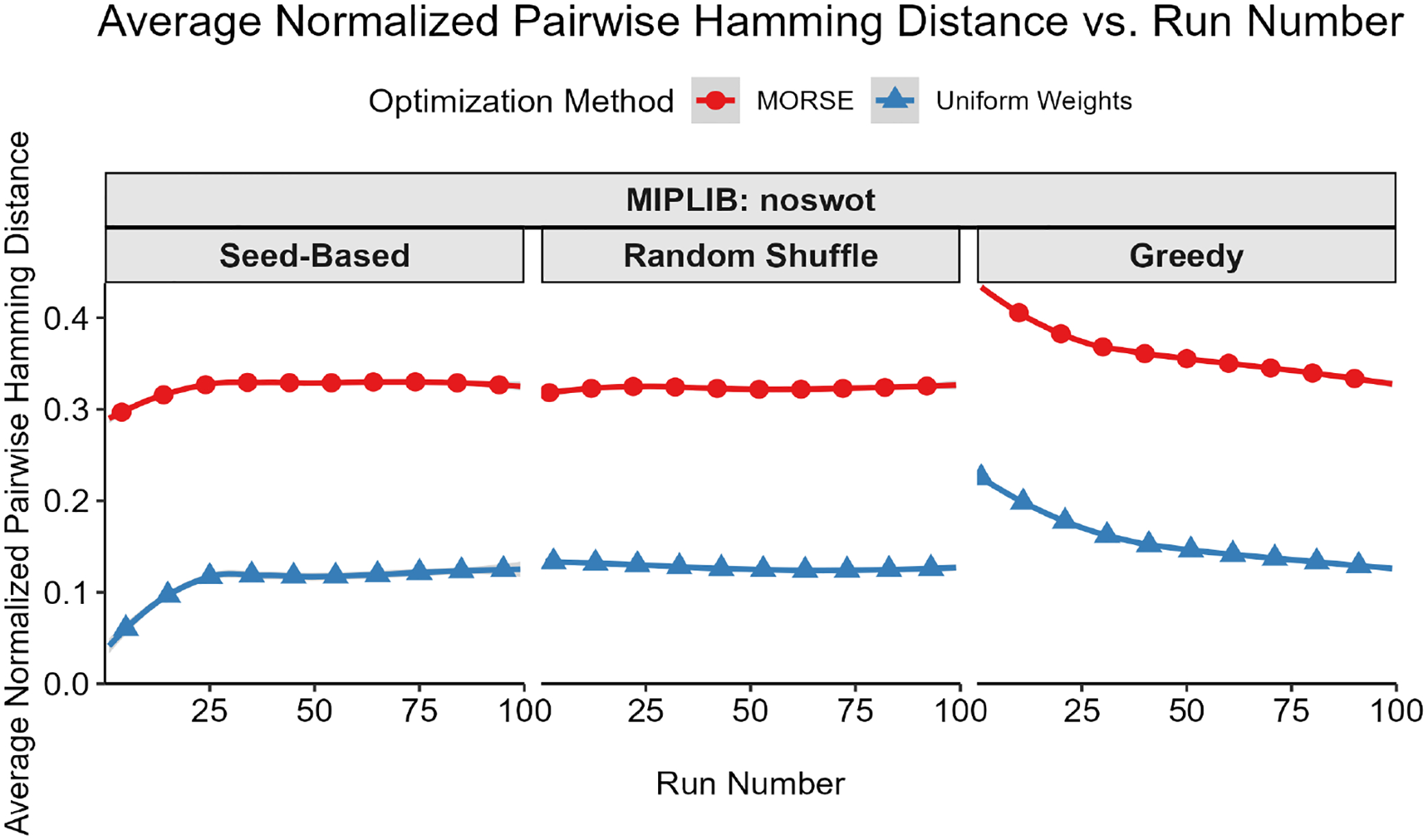
Average normalized pairwise Hamming distance (*y*-axis) vs. run number (x-axis) for MIPLIB instance noswot.

**Figure 2.**
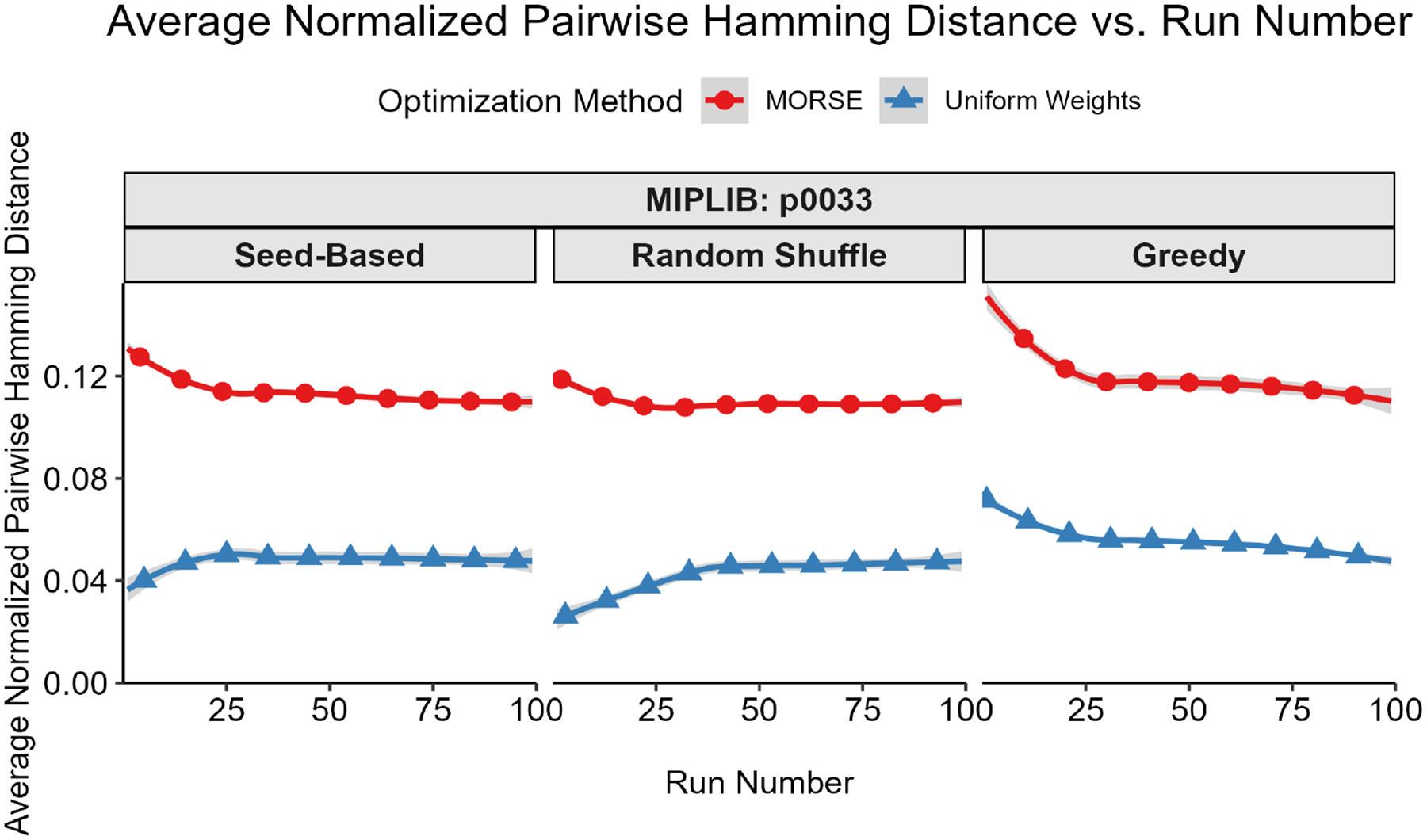
Average normalized pairwise Hamming distance (y-axis) vs. run number (x-axis) for MIPLIB instance p0033.

**Figure 3.**
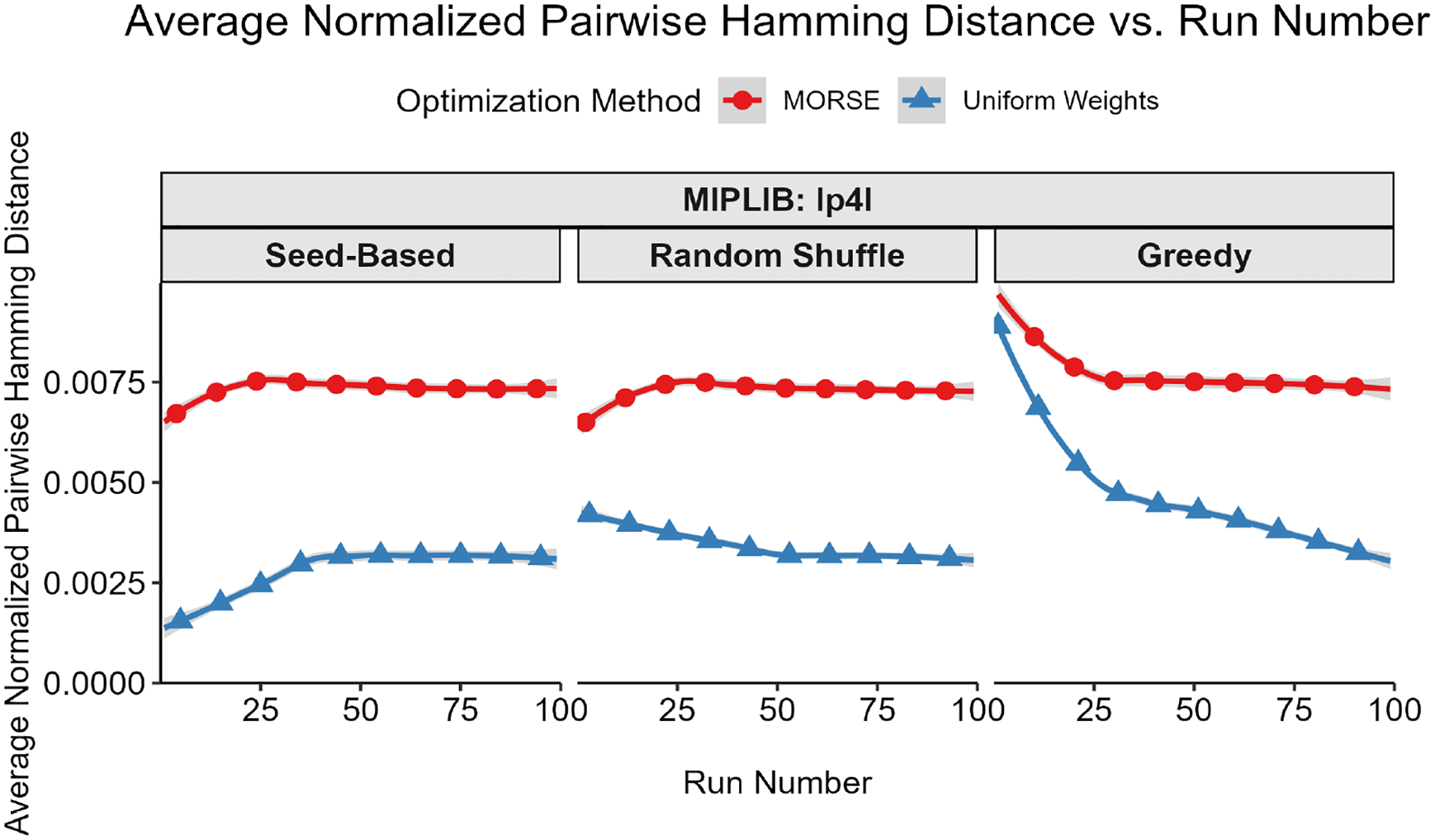
Average normalized pairwise Hamming distance (y-axis) vs. run number (x-axis) for MIPLIB instance lp4l.

**Figure 4.**
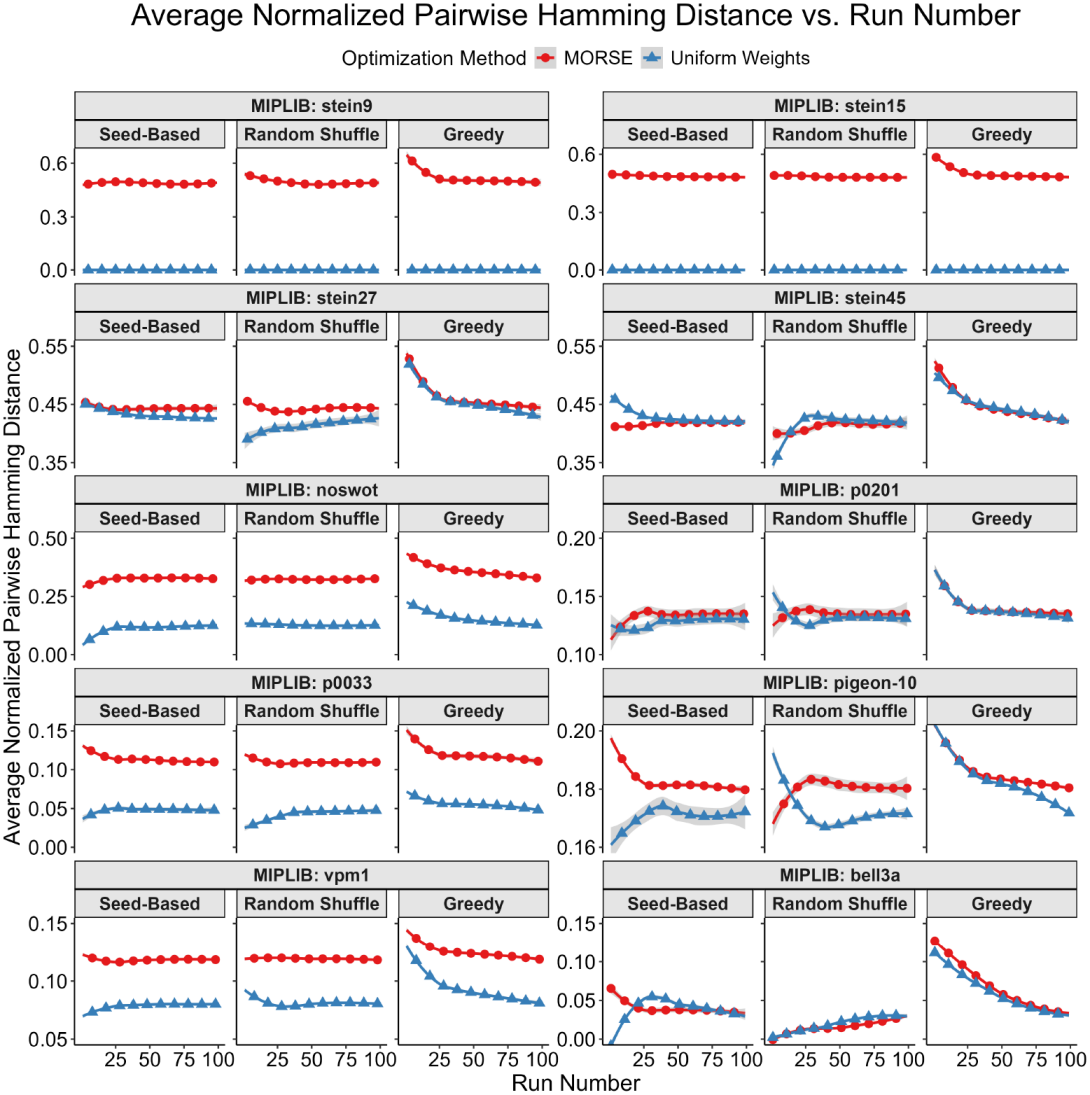
Tabular results. Average normalized pairwise Hamming distance (y-axis) vs. run number (x-axis) for tested MIPLIB instances with the 10 highest maximum average normalized pairwise Hamming distances. The plots are arranged in descending order of maximum average normalized pairwise Hamming distance. Please note that the y-axis starts above 0 for some panels.

**Figure 5.**
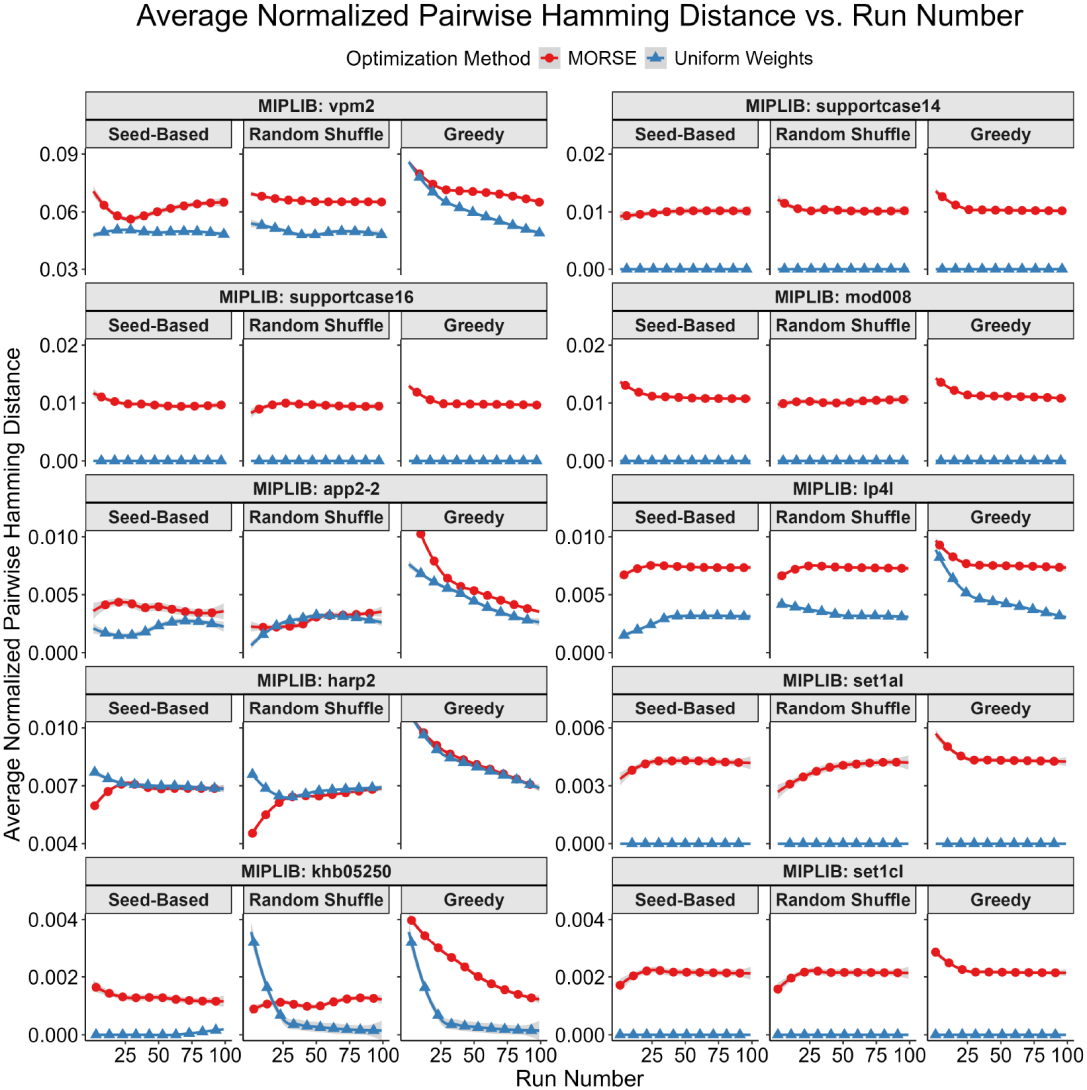
Tabular results. Average normalized pairwise Hamming distance (y-axis) vs. run number (x-axis) for tested MIPLIB instances with the 10 lowest maximum average normalized pairwise Hamming distances. The plots are arranged in descending order of maximum average normalized pairwise Hamming distance. Please note that the y-axis starts above 0 for some panels.

Our first test measured diversity as quantified by the Hamming distances (see Definition 1) for binary and integer variables between pairs of optima selected. To account for potential variations in integer representations, we rounded each binary/integer variable to the nearest integer. For each instance, we built a plot of average pairwise Hamming distance normalized by the total binary solution size (y-axis) vs. solution number (x-axis) for both MORSE and uniform weights for each of the three methods described above. For every sorting method, the average pairwise Hamming distance between optima found with MORSE exceeded the pairwise Hamming distance of optima found with the uniform weights method at almost all run numbers (see Figures 1–5 below). The shaded gray area surrounding the lines gives the 95% confidence interval.

The second test measured the number of distinct optima found as a function of run number. We formally define “distinct” optimal solutions as those that differ on variables in the objective function; two solutions with identical objective function variable values, but different non-objective function variable values, are considered identical for this test. The motivation for focusing on the objective function variables is that they are the variables of interest in applications such as MadHitter. If each optimum has an equal probability of being found (see Question 3 above), then the expected number of runs s needed to find all optima grows as slog(s) ([45] Section 2.4, pp. 32–33). For the second test, we executed r runs with MORSE and r runs with uniform weights and collected 1 optimum for each run. The value of r ranged from 100 to 2000, depending on the number of distinct optima for a given instance.

**Figure 6.**
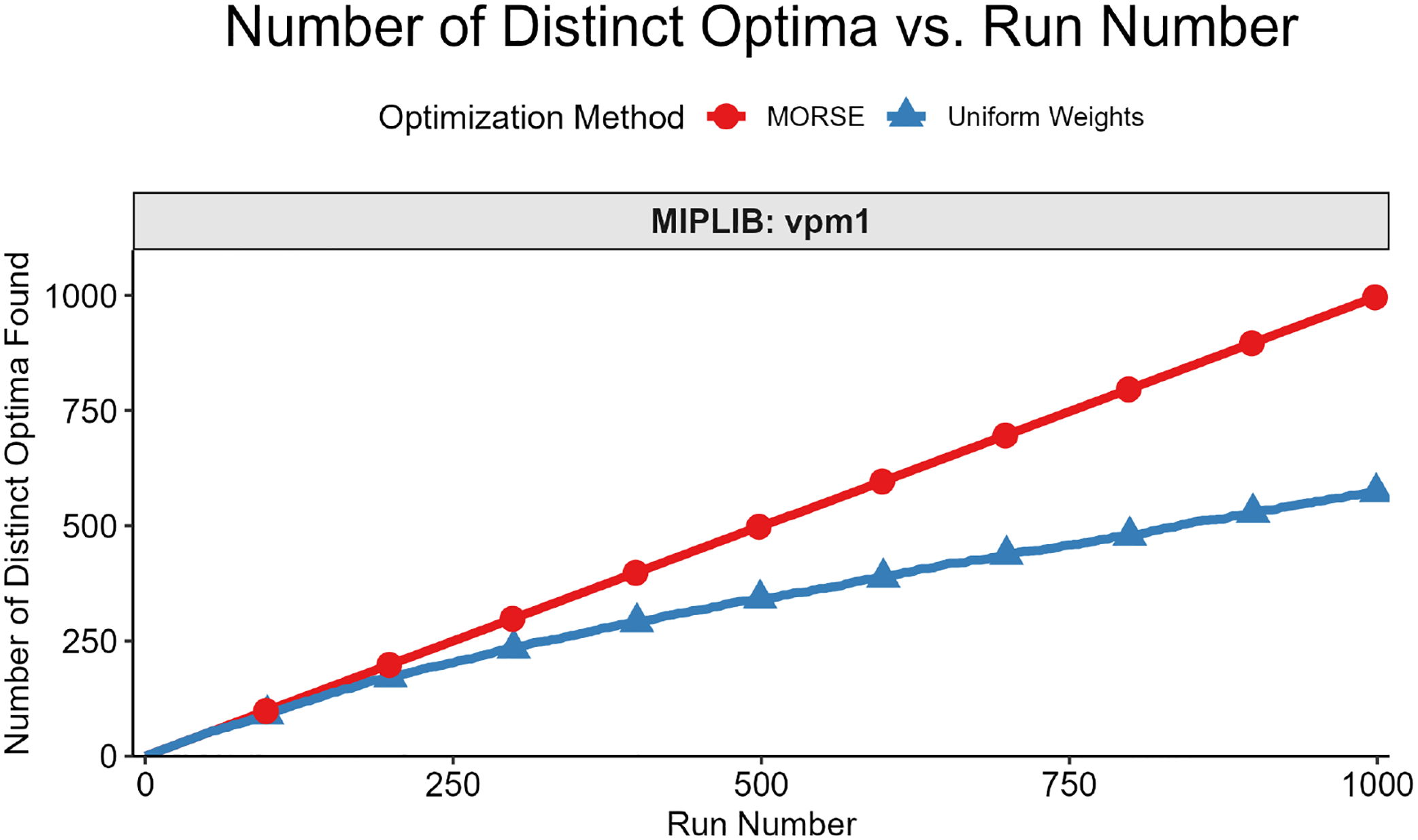
Number of distinct optima vs. run number for MIPLIB instance vpm1. While we cannot find all optima in the allotted 1000 runs, we find 998 distinct optima with MORSE and 573 distinct optima with the uniform weights method.

**Figure 7.**
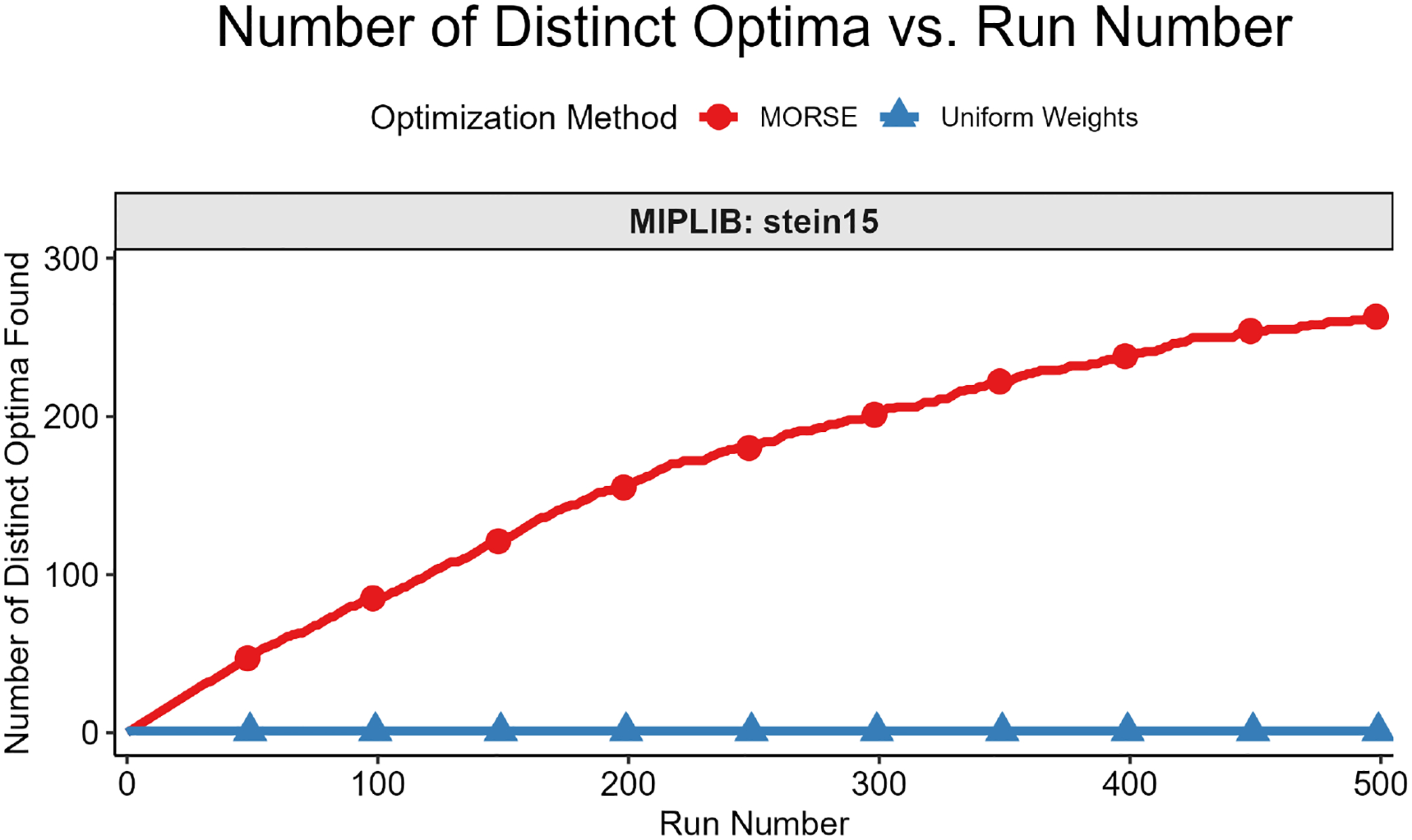
Number of distinct optima vs. run number for MIPLIB instance stein15. With MORSE, we find 264 distinct optima in the allotted 500 runs, while we find one optimum with the uniform weights method.

**Figure 8.**
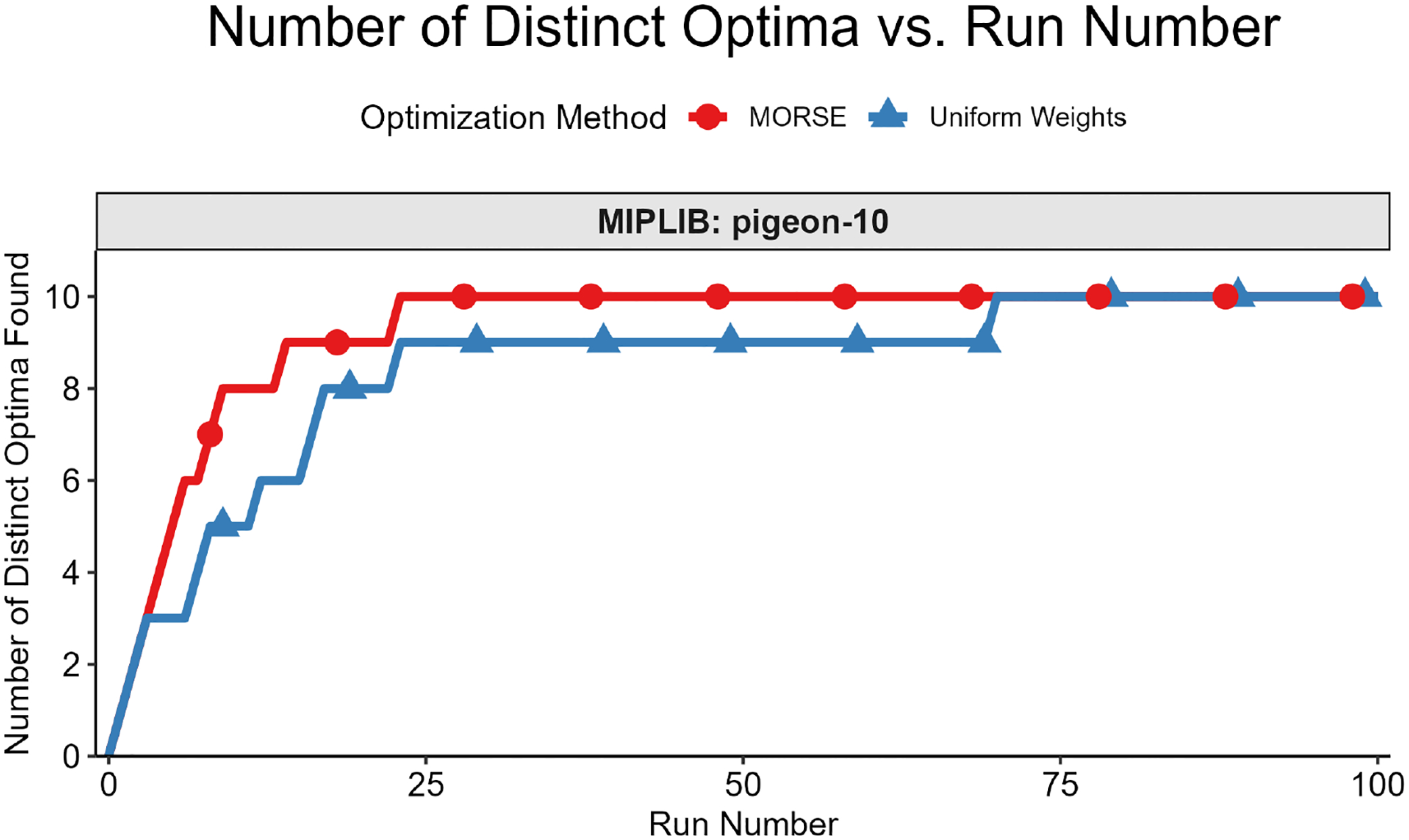
Number of Distinct Optima vs. Run Number for MIPLIB instance pigeon-10. With MORSE, we find 10 distinct optima in 23 runs, while 70 runs are needed to find 10 distinct optima using the uniform weights method.

We then plotted the number of distinct optima (y-axis) found vs. run number (x-axis). This test was best suited for instances with <100 distinct optima, all of which we were able to find in a reasonable number of runs, thus allowing us to compare the performance of the two weights methods in finding distinct optima. Each test result fit one of the two following scenarios:

1. While we cannot find all optima in the allotted r runs, we observe that we find more optima with MORSE than with the uniform weights method. (see Figures 6 and 7)
2. We find the same number of optima in the allotted r runs with both methods, but fewer runs are needed with MORSE than with the uniform weights method (see Figure 8)

**Figure 9.**
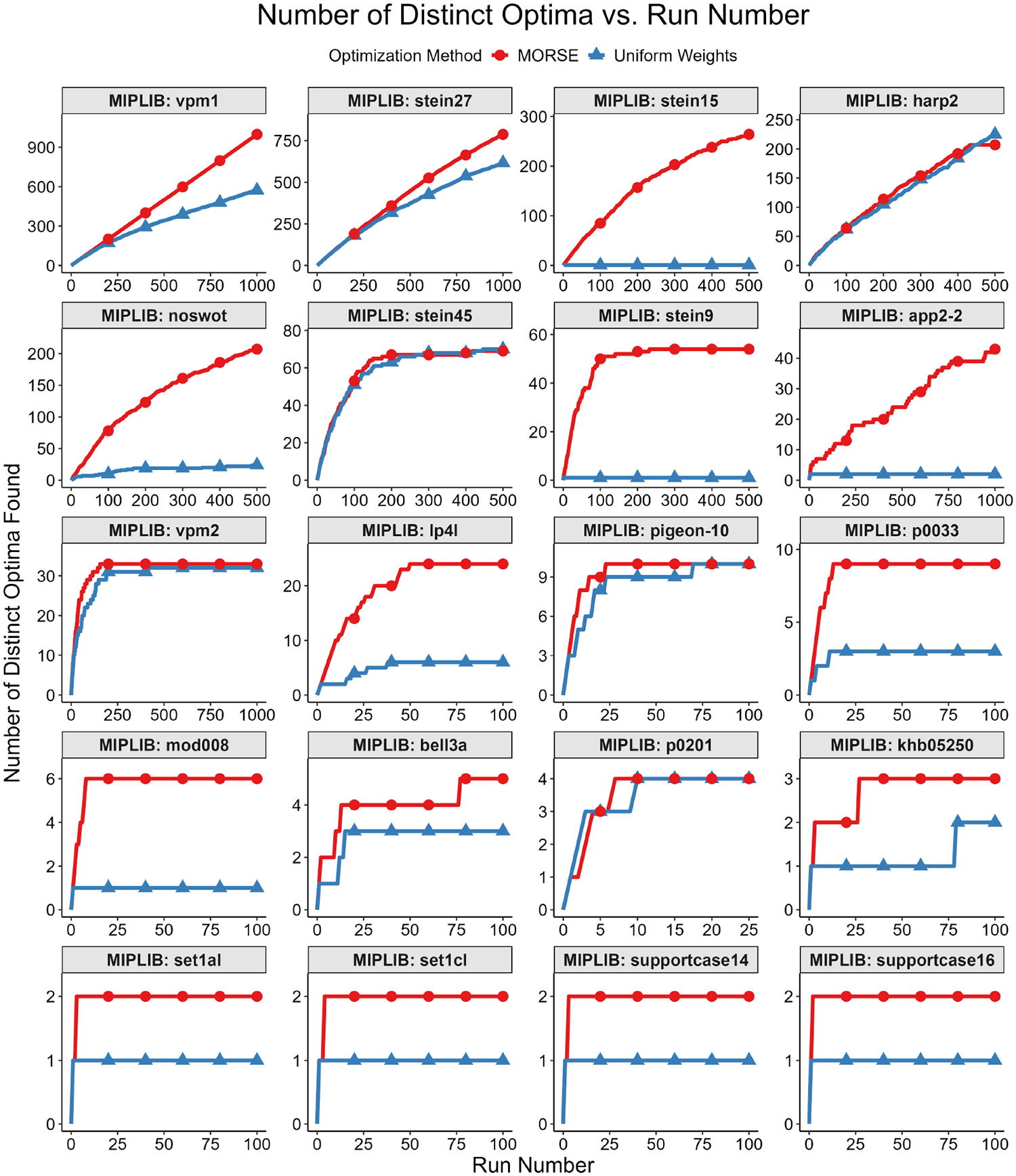
Full MIPLIB results: number of distinct optima vs. run number for all MIPLIB instances tested. The plots are arranged in descending order of maximum number of unique optima found.

Both numbered scenarios favor MORSE, so we conclude empirically that our method performs better than the homogeneous weights method on the second test. For full MIPLIB results, see Figure 9.

We tested the method of [4] as implemented in the CPLEX populate function on the 15 instances in Table 1 for which the exact number of distinct optima is known. We initially set the PopulateLim parameter, which controls the maximum number of solutions returned for the python interface, to the known number of optima for each instance. With that setting, CPLEX found all optima for 12/15 instances, but only found 1 out of 2 known solutions for the instances set1al and set1c1, as well as 1 out of 51 solutions for the instance khb05250. With PopulateLim set to higher values, CPLEX found the second solutions for set1al and set1cl but did not find more than 1 solution for khb05250 (Table 2). We conclude that this method works well on most instances if one knows the number of solutions beforehand (as is rarely the case in most practical settings), but does not work perfectly.

In addition to testing MORSE on MIPLIB instances, we tested on instances from MadHitter, an algorithm that identifies gene targets for experimental CAR-T, conjugated antibody, and coated nanoparticle therapies [1]. The algorithm takes single-cell tumor data and non-tumor data for a set of patients and returns the smallest set of target genes that kills at least a specified *lower bound* percentage of each patient’s tumor cells (for example, ≥80%) while killing at most a specified *upper bound* percentage of each patient’s non-tumor cells (for example, ≤10%). The single-cell datasets were reduced to focus on the 1269 genes encoding cell surface receptors, which may serve as targets for one or multiple of the experimental therapies listed above [1]. The objective is to minimize the number of treatments that a patient must receive. In a second layer of optimization for a cohort of multiple patients, we optimize the total number of distinct gene targets used, such that the number of targets for each patient is optimal. Intuitively, if a single patient has multiple sets of gene targets of the same optimal size, we select the optimal set that best permits us to reuse the same targets for other patients in the cohort; the exact ILP formulation is given in [1].

For each feasible instance (dataset, lower bound, and upper bound permutation for which an optimum exists), we executed 1000 runs for both MORSE (in which each gene is randomly assigned a weight in the range [1-ε,1+ε]) and the uniform weights method (in which each gene has a uniform weight of 1). Since all solutions found were composed of fewer than 100 targets, ε=0.01 was used for all instances. This value of ε was chosen such that the maximum magnitude of the sum of perturbations, given by ε⋅(*number of targets in solution*), did not exceed 1.

**Figure 10.**
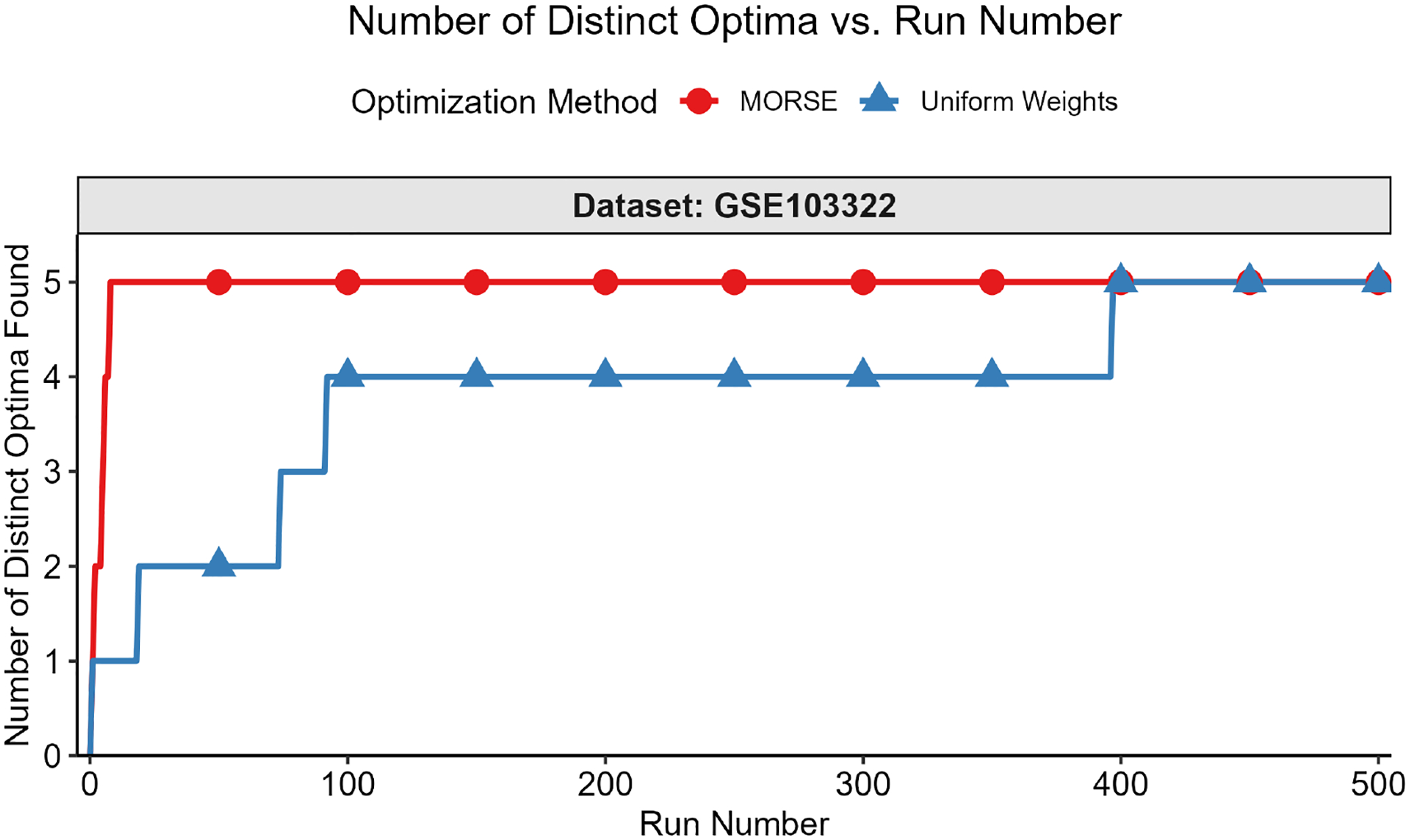
Number of distinct optima found (y-axis) vs. run number (x-axis) for GSE103322 (brain cancer) 1269-gene dataset, lower bound = 0.8, upper bound = 0.1. With MORSE, we find all 5 distinct optima in 9 runs. With uniform weights, we find these optima in 397 runs.

**Figure 11.**
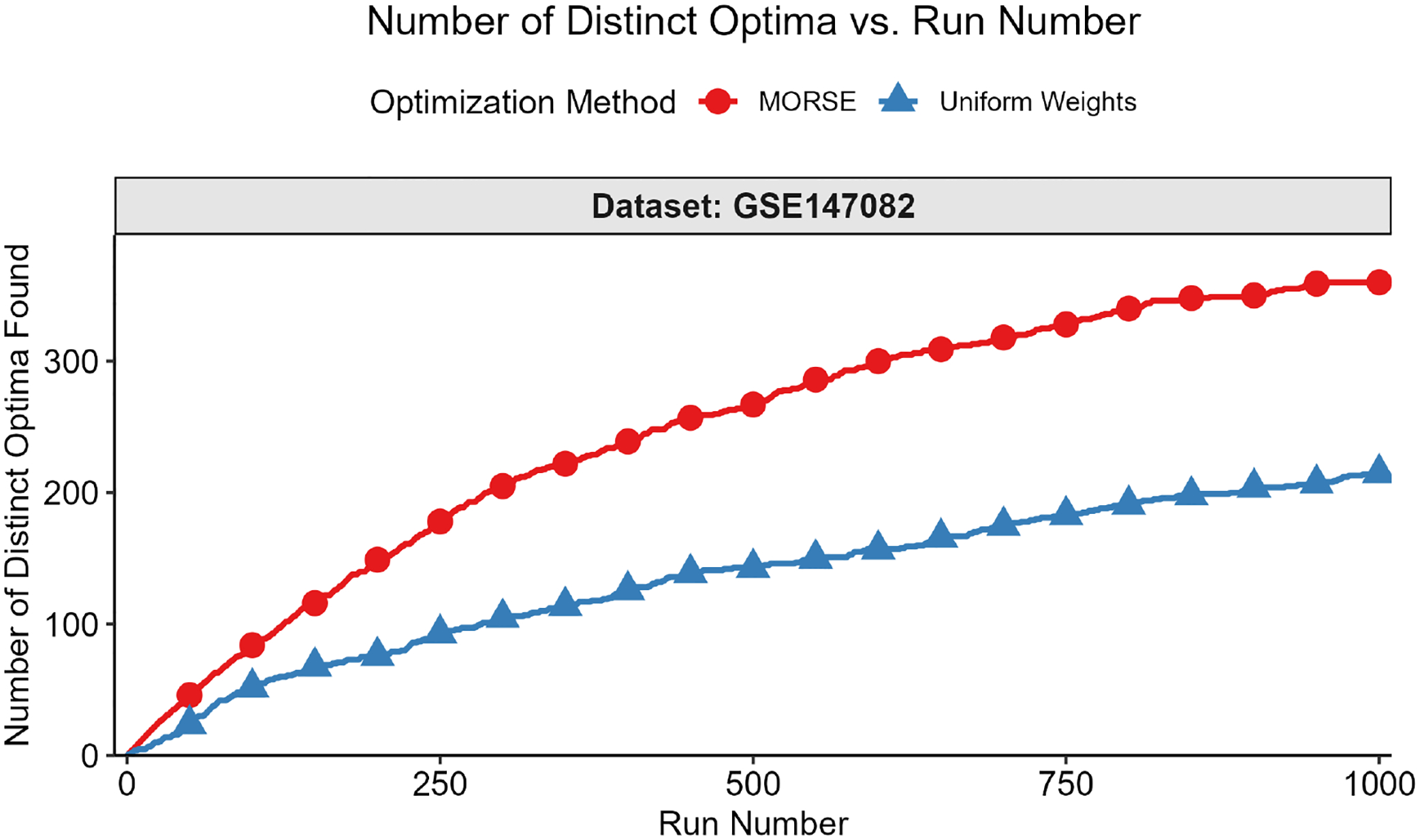
Number of distinct optima found (y-axis) vs. run number (x-axis) for GSE147082 (ovarian cancer) 1269-gene dataset, lower bound = 0.8, upper bound = 0.1. With MORSE, we find all 360 distinct optima in 959 runs. With uniform weights, we find 215 optima in 1000 runs.

**Figure 12.**
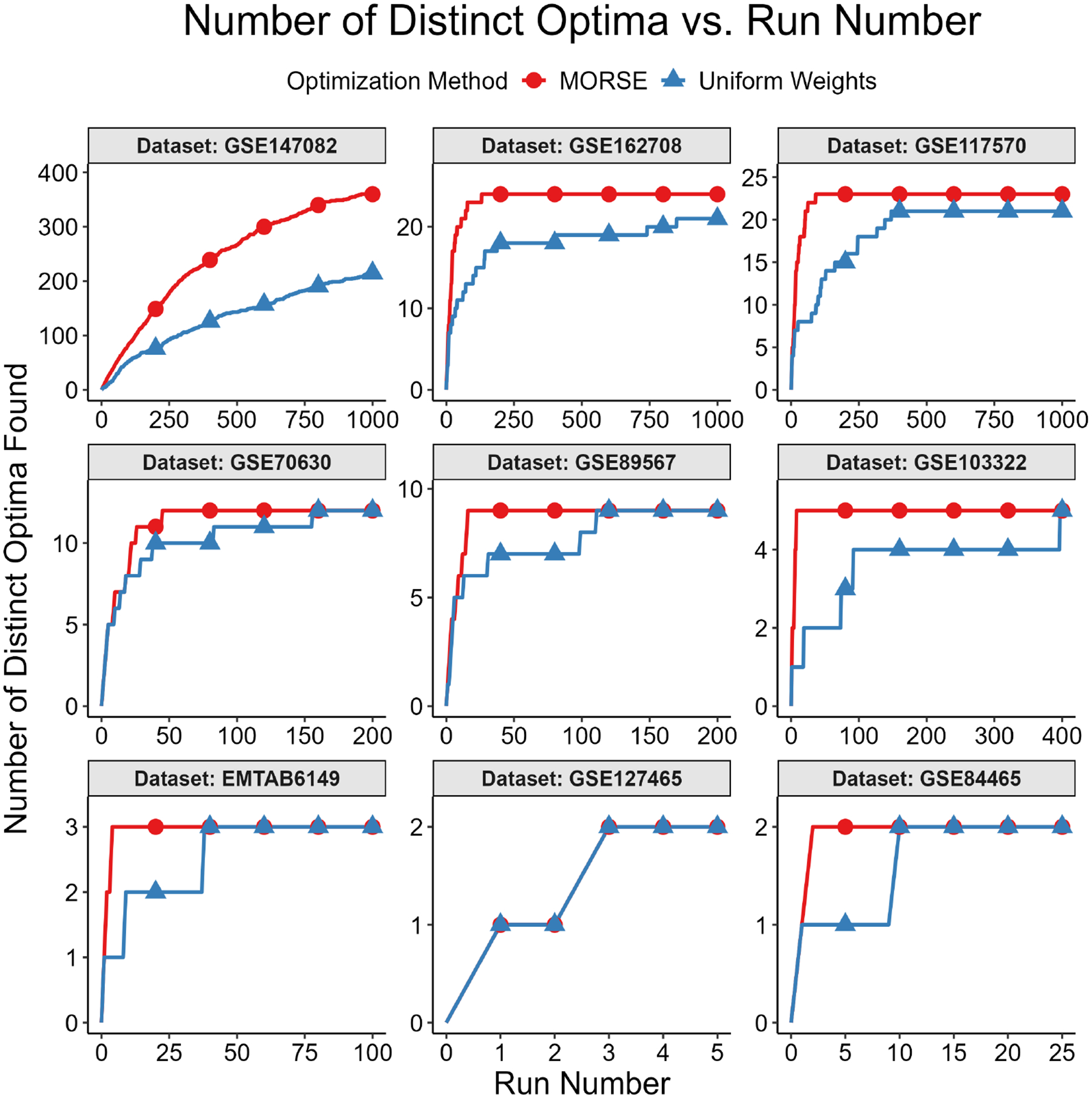
Tabular results. Number of distinct optima found (y-axis) vs. run number (x-axis) for all 1269-gene cancer datasets tested, lower bound = 0.8, upper bound = 0.1. For GSE127465 (lower row, center), the results for MORSE (red) and uniform weights (blue) are identical

Just as we did for the MIPLIB instances, we measured the number of distinct optima found as a function of run number. Distinct solutions were defined as solutions that differed globally; we did not account for differences in local (patient) variables. For example, consider *global optimum* A, given by [G1, G2, G3], with corresponding local optima as follows: *patient 1* receives the treatments [G1, G2] and *patient 2* receives the treatments [G2, G3]. Next, consider global optimum B, also given by [G1, G2, G3], with corresponding local optima as follows: *patient 1* receives the treatments [G1, G3], and *patient 2* receives the treatments [G1, G2]. Global optimum A and global optimum B are considered identical for the purposes of this test. Results with many choices for the number of runs on the x-axis and two individual datasets can be found in Figures 10 and 11. Results with fewer choices for the number of runs for nine datasets can be found in Figure 12.

A practical motivation for looking at multiple optima of MadHitter instances is to identify which genes occur in any optimal solution or in all optima. An observed example of the impact of MORSE is conveyed by analyzing the brain cancer dataset GSE103322. With default MadHitter parameters and using the solution listing delineated above, the gene *CELSR1* (short for cadherin EGF LAG seven-pass G-type receptor 1) appears (by chance) in multiple optima generated by MORSE, including the second optimum. However, when using Gurobi’s solution pool, *CELSR1* appears for the first time in optimum number 397. The gene *CELSR1* has previously been shown to have higher messenger RNA levels in brain cancer as compared to surrounding non-cancerous brain tissue by [46,47], so this is a favorable gene to select as a possible target in an optimal treatment.

**Figure 13.**
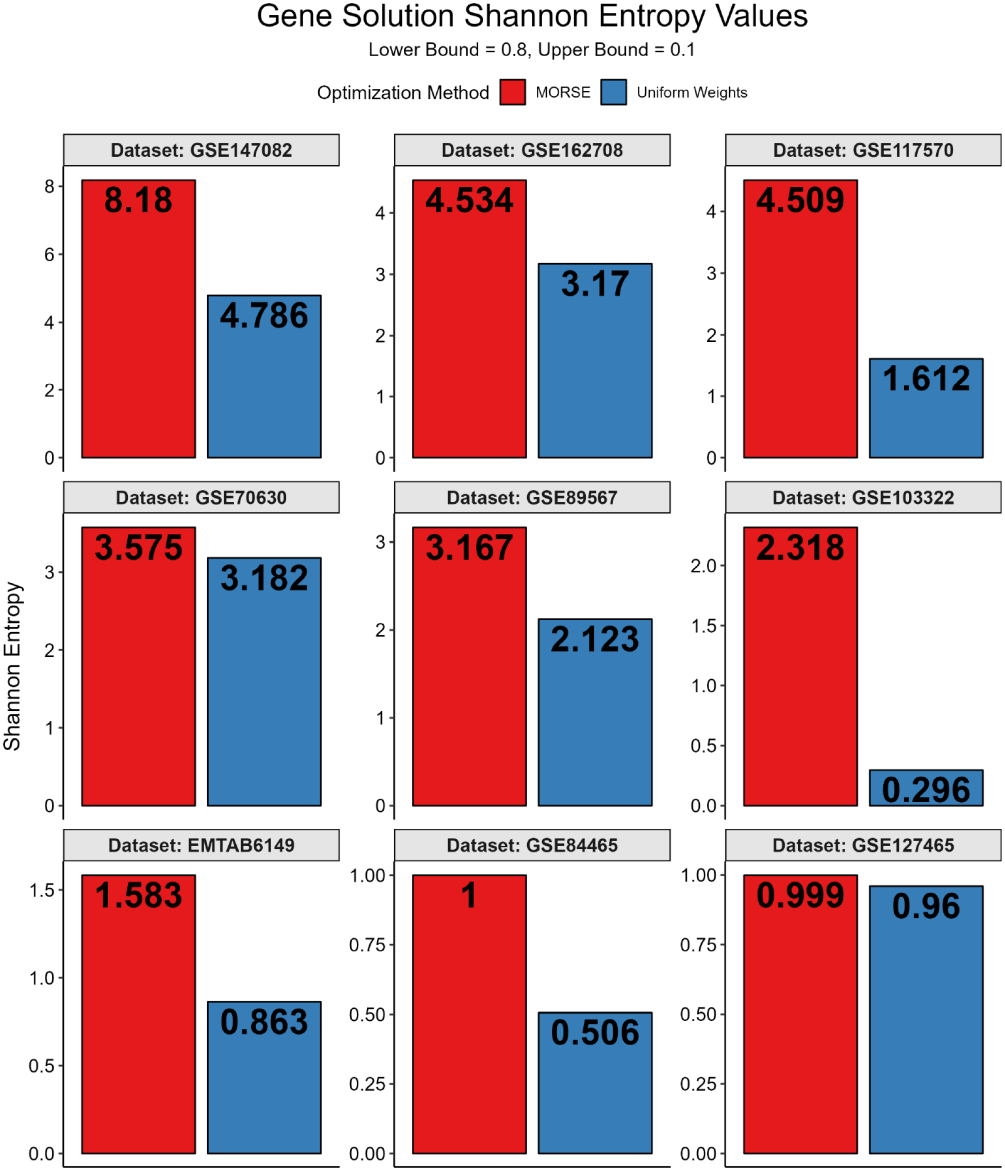
Gene solution Shannon entropy values for datasets with feasible solutions at lower bound = 0.8, upper bound = 0.1. The plots are arranged in descending order of the maximum Shannon entropy. The Shannon entropy for MORSE runs exceeds that of uniform weights runs for all datasets used.

To quantify bias in the solution space, we used Shannon entropy. A higher Shannon entropy value corresponds to a higher level of randomness and a lower level of bias in the distribution. In the present context, a high Shannon entropy value is desirable, as the algorithm should not favor any particular solution in the solution space. To find the Shannon entropy for each feasible permutation, we assigned an empirical normalized probability $p_{i}$ to each gene solution in the solution set and applied the standard Shannon entropy formula (see Section 2.7). Full MadHitter Shannon entropy results can be found in Figure 13.

**Figure 14.**
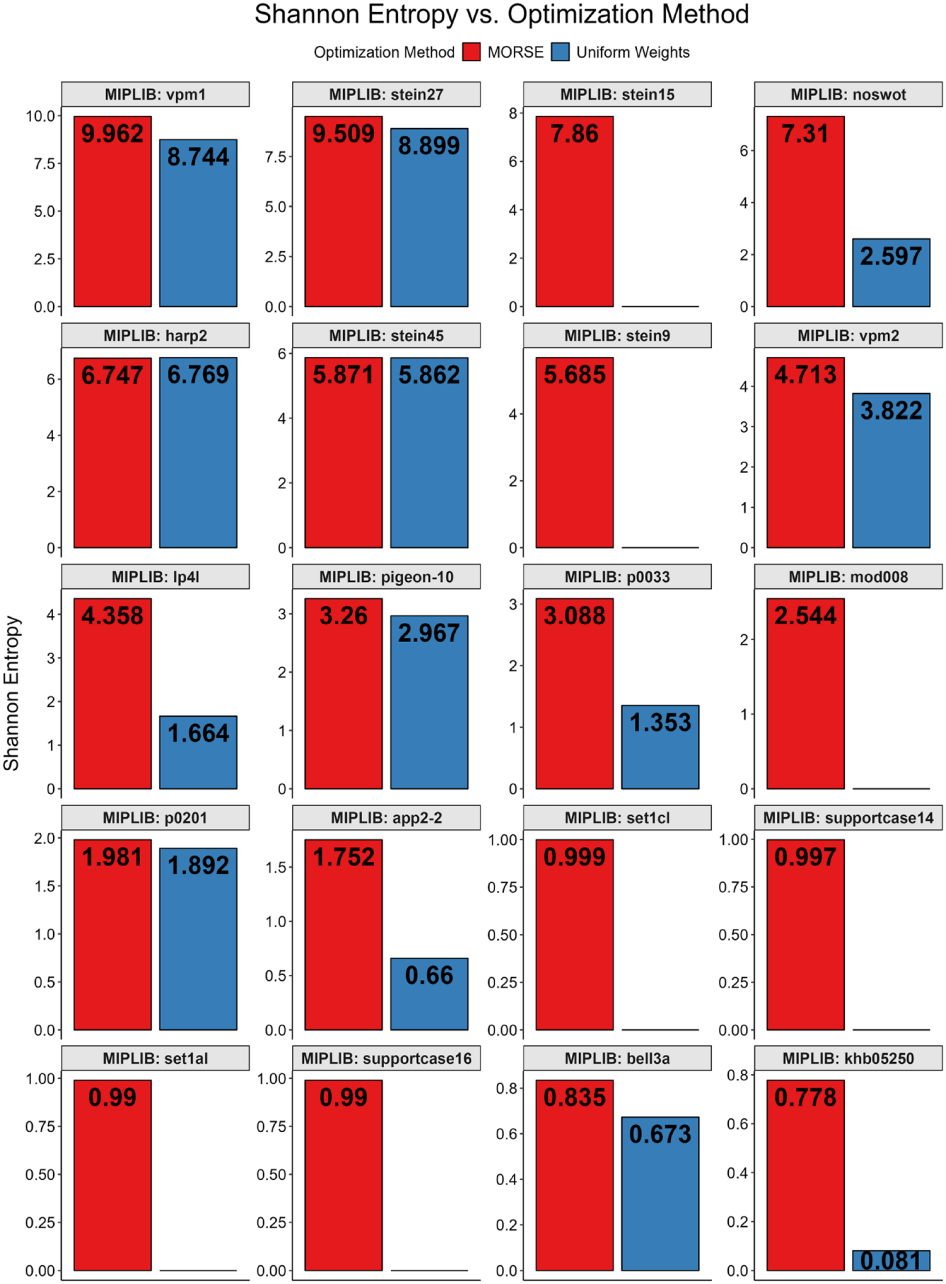
Full MIPLIB results, Shannon entropy vs. optimization method for all MIPLIB instances tested. The plots are arranged in descending order of maximum Shannon entropy.

**Figure 15.**
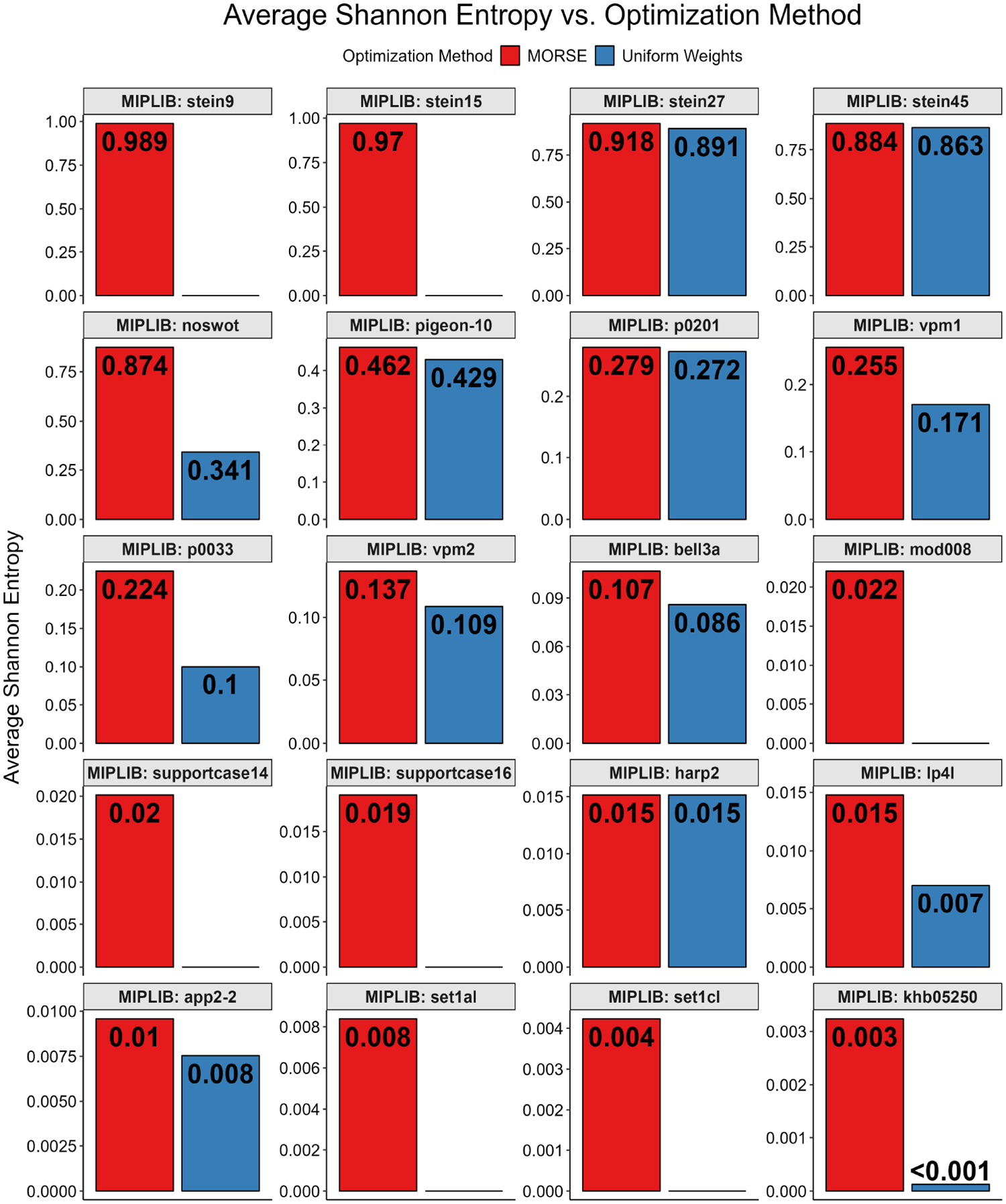
Full MIPLIB results, average Shannon entropy vs. optimization method for all MIPLIB instances tested. The plots are arranged in descending order of maximum average Shannon entropy.

For the MIPLIB instances tested, we also employed the Shannon entropy on the optima found with both MORSE and the uniform weights method. We calculated the Shannon entropy on the multiplicity of each optimum found (Figure 14) by assigning an empirical normalized probability $p_{i}$ to each distinct optimum found. Additionally, for each instance tested, we calculated Shannon entropy on each variable by assigning $p_{i}$ to each distinct variable value, then averaged the Shannon entropy score for each variable to find the average Shannon entropy (Figure 15). Both tests only considered the variables in the objective function. We found that, for both approaches, the Shannon entropies for optima found with MORSE generally exceeded those of the optima found with uniform weights, except for one instance (harp2) where the latter marginally exceeded the former.

When computing Shannon entropies over variables and averaging, we assign more influence to variables whose values differ more often between different optimal solutions. In practical applications, it may be useful to consider whether the objective function terms or constraints involving the high-entropy variables should be refined to favor one or some of the possible values, e.g., if a high-entropy variable has a positive coefficient in the objective function of a minimization problem (as in MIPLIB), then one could favor higher positive values for the variable by decreasing its coefficient. At the lower extreme, a variable that takes on the same value in every optimal solution contributes a Shannon entropy of 0 and it may be less useful to change the terms involving that no-entropy variable in the problem instance.

## 4. Discussion

We implemented and evaluated MORSE, a perturbation method to find multiple optima for ILPs. After [33], few subsequent papers have investigated randomization in this context (see Section 1.2). A key advantage of the present work and other randomized methods is that they are amenable to parallelization. Another distinguishing feature is that we proved that our perturbation method preserves optima when all the coefficients are integers, which Trapp and Konrad did not prove for their implementation of a randomized algorithm ([6]).

With MORSE, we generally find more diverse optima (as quantified by higher average pairwise Hamming distance between solution vectors) within the first 100 distinct optima returned for MIPLIB instances with >100 optima. Additionally, when executing n runs in parallel, we find distinct optima at a faster rate with MORSE than with the method in Gurobi. Based on the number of optima and the number of runs allotted for a given instance, we either find the same number of optima in fewer runs or find a greater number of optima using MORSE. An important practical application of our method is to decide quickly if an instance has more than one optimal solution. For example, in Figure 9, subpanels for supportcase14 and supportcase16, we see two instances in which Gurobi’s solution pool method suggests that the optimal solution is unique, but MORSE finds two distinct optimal solutions. Similarly, the CPLEX populate function returned just one optimum each for the MIPLIB instances set1a1 and set1c1, despite setting the parameter values to return both optima. Moreover, the SCIP count function requires multiple steps to decide if there is more than one optimal solution: first solving to optimality, and then constructing and solving a modified ILP.

We also integrated our method with MadHitter, an algorithm that identifies minimal gene target hitting sets for experimental gene therapy cancer treatments by formulating the problem as an ILP. Similar to the result we obtained when testing on MIPLIB instances, we either found all optimal gene target sets with MORSE, or a greater number of optimal gene target sets, after executing 1000 runs in parallel compared to Gurobi. We quantified the Shannon entropy of each vector of 1000 runs for each dataset and weights method tested, and found that, for any given dataset, the Shannon entropy for MORSE runs exceeded that of uniform weights runs, indicating a higher diversity among the optima found by MORSE.

### 4.1. Limitations

The proposed method is not applicable to MIP problems in which all of the variables in the objective function are continuous, nor is it applicable to (continuous) linear programming problems, in which all decision variables may take on continuous values. A key limitation is that the MORSE method does not guarantee finding all optima, even for ILPs. It is also possible that the scaling procedure could produce excessively large objective function coefficients, leading to numerical instability in the optimization solver. In our testing, this issue did not occur, as we verified that all solutions returned were optimal.

An additional limitation of the proposed method is that it is less amenable to computing environments in which parallel computing is not supported, or in which doing p>1 runs in parallel costs substantially more than one run in whatever currency (e.g., money or computing allocation) is used to discourage users from executing many runs in parallel. In the SLURM-based computing environments (at the National Institutes of Health and Northwestern University) in which we conducted testing, submitting several runs in parallel is only mildly discouraged by a reduction in priority for jobs, so our highly parallelized method works well. For a given instance, each run of MORSE with a different random seed can be (and typically was) queued simultaneously to be executed in parallel. We could not accurately measure the subsequent speedup, as the computing environments used in testing were shared with hundreds of other users, and any degradation from perfect speedup depended on the competing non-MORSE jobs in the queue.

### 4.2. Future Directions

Given the desire of biologists and biochemists to find all optima to problems of interest, the present algorithm could be applied to domain-specific instances in the field of biology, such as haplotype inference [7] and other applications listed in Section 1. There also exists the possibility of developing other domain-specific sampling schemes, naturally leading to more extensive explorations of the permutation hypercube described in Section 2.4.

Other relevant recent studies, for example by [48–51], focus on finding a collection of k solutions that are approximately optimal and maximizing their diversity (measured by the sum of distances between solutions). The underlying optimization problems may be either in P or are NP-hard, and these studies connect the search for multiple solutions to other algorithmic concepts, such as dynamic programming dispersion and fixed-parameter tractability. Their goal is not necessarily to find all optimal solutions, but instead to find a pre-specified number of high-quality, diverse solutions. Accordingly, explicitly optimizing diversity of the solutions produced could be an interesting future direction for our work as well, especially on instances with a very large collection of optimal or near-optimal solutions.

## 5. Conclusions

We present MORSE (Multiple Optima via Random Sampling and careful choice of the parameter Epsilon), a randomized algorithm designed to efficiently generate multiple optimal solutions for Integer Linear Programs (ILPs) and Mixed Integer Programs (MIPs). MORSE maps random multiplicative perturbations to the coefficients on binary/integer objective function variables, breaking ties among optimal solutions and enabling the identification of distinct optima. The algorithm is parallelizable, making it well-suited for modern computing environments and addressing the shortcomings of the existing, inherently sequential methods for the identification of multiple optima. Empirical results demonstrated that MORSE outperforms the Gurobi solution pool method as measured by the number of distinct optima found and the diversity of solutions. MORSE also exhibited superior performance on MadHitter, a practical application in cancer genomics for which the identification of multiple optimal gene target sets is crucial. The algorithm’s ability to quickly identify diverse solutions makes it particularly valuable in fields in which the analysis of multiple optima can provide critical domain-specific insights, such as biomedicine.

## Figures and Tables

**Table 1.** List of tested MIPLIB instances, the highest-numbered version of MIPLIB in which each instance appears, and the corresponding number of distinct optima. MIPLIB versions 2.0 and 3.0 were released in 1996, version 4.0 was released in 2003, version 5.0 was released in 2010, and version 6.0 was released in 2017. Distinct optima were enumerated using the PySCIPOpt count function [3]. Instances marked with * are also found in Table 1 of [4], and the number of optima claimed in that table is consistent with the number of optima found by count. Distinct optima counts marked with ≥ give the number of optima enumerated by count within a 24 h runtime; if there is no ≥, then enumeration was fully completed in runtime < 24 h. We did not perform full MORSE testing on any MIPLIB instance not listed in this table; some instances were excluded by the preliminary testing described in Section 2.6. The criteria for choosing the instances are summarized in Section 2.6

MIPLIB Instance	Most Recent MIPLIB Version	Number of Distinct Optima
set1al	2.0	2
set1cl	2.0	2
p0201	3.0	* 4
bell3a	3.0	5
mod008	3.0	* 6
p0033	3.0	* 9
lp41	2.0	24
vpm2	4.0	33
khb05250	3.0	51
stein9	2.0	54
stein45	3.0	* 70
supportcase14	6.0	256
supportcase16	6.0	256
stein15	3.0	315
stein27	3.0	* 2106
harp2	5.0	≥1
pigeon-10	6.0	≥6574
app2-2	6.0	≥41,819
noswot	6.0	≥48,776
vpm1	3.0	≥86,186

**Table 2.** Tested MIPLIB instances for which the CPLEX populate function did not find all optimal solutions.

MIPLIB Instance	PopulateLim Setting	Number of Distinct Optima (CPLEX)	Number of Distinct Optima (SCIP)	Number of Missed Optima
khb05250	51	1	51	50
khb05250	102	1	51	50
khb05250	204	1	51	50
khb05250	408	1	51	50
khb05250	816	1	51	50
khb05250	1632	1	51	50
khb05250	3264	1	51	50
khb05250	6528	1	51	50
khb05250	13,056	1	51	50
khb05250	26,112	1	51	50
set1al	2	1	2	1
set1cl	2	1	2	1

## Data Availability

The MORSE algorithmic code can be found at https://github.com/ruppinlab/MORSE (accessed on 2 March 2025). The input files, scripts, and testing data used to produce the results/figures presented in this manuscript can be found at https://github.com/ruppinlab/MORSE/tree/main/MORSE_data (accessed on 2 March 2025).

## Abbreviations

The following abbreviations are used in this manuscript:

ILP: integer linear program
MIP: mixed integer (linear) program
MORSE: Multiple Optima via Random Sampling and careful choice of the parameter Epsilon
